# Supplementary material for: Association between malignancies and Marfan syndrome: a population-based, nested case–control study in Taiwan
Source: BMJ Open. 2017 Oct 16;7(10):e017243. doi: 10.1136/bmjopen-2017-017243 (PMC5652471; doi:10.1136/bmjopen-2017-017243)
Supplement: Author's manuscript [file bmjopen-2017-017243.draft_revisions.pdf]

BMJ Open is committed to open peer review. As part of this commitment we make the peer review history of every article we publish publicly available.

When an article is published we post the peer reviewers' comments and the authors' responses online. We also post the versions of the paper that were used during peer review. These are the versions that the peer review comments apply to.

The versions of the paper that follow are the versions that were submitted during the peer review process. They are not the versions of record or the final published versions. They should not be cited or distributed as the published version of this manuscript.

BMJ Open is an open access journal and the full, final, typeset and author-corrected version of record of the manuscript is available on our site with no access controls, subscription charges or pay-per-view fees (http://bmjopen.bmj.com).

If you have any questions on BMJ Open's open peer review process please email editorial.bmjopen@bmj.com

BMJ Open

Increased risk of malignancies in patients with Marfan syndrome: a nationwide, population-based, nested case-control study

Journal:	*BMJ Open*
Manuscript ID	bmjopen-2017-017243
Article Type:	Research
Date Submitted by the Author:	10-Apr-2017
Complete List of Authors:	Hsu, Chin-Wang ; School of Medicine, College of Medicine, Taipei Medical University, Department of Emergency Medicine,<br>Wang, Jen-Chun; Tri-Service General Hospital, National Defensive Medical Center, department of Emergency Medicine<br>Liao, Wen-I; Emergency Medicine<br>Chien, Wu-Chien ; National Defense Medical Center<br>Chung, Chi-Hsiang; National Defense Medical Center<br>Tsao, Chang-Huei ; Tri-Service General Hospital, National Defensive Medical Center, department of Emergency Medicine, Department of Medical Research<br>Wu , Yung-Fu ; Tri-Service General Hospital, National Defense Medical Center<br>Liao, Min-Tser ; Taoyuan Armed Forces General Hospital,<br>Tsai, Shih-Hung; Tri-Service General Hospital, National Defense Medical Center, Department of Emeregncy Medicine
**Primary Subject Heading**:	Oncology
Secondary Subject Heading:	Genetics and genomics, Oncology
Keywords:	Marfan syndrome, malignancy, transforming growth factor-β, fibrillin

SCHOLARONE™ Manuscripts

For peer review only - http://bmjopen.bmj.com/site/about/guidelines.xhtml

Increased risk of malignancies in patients with Marfan syndrome: a nationwide, population-based, nested case-control study

Chin-Wang Hsu MD [1,2], Jen-Chun Wang MD [3,4], Wen-I Liao MD [3], Wu-Chien Chien PhD [5,6,*], Chi-Hsiang Chung PhD [5,6,7], Chang-Huei Tsao PhD [5], Yung-Fu Wu PhD [5], Min-Tser Liao, MD[8], Shih-Hung Tsai MD, PhD [3,*]

[1] Department of Emergency Medicine, School of Medicine, College of Medicine, Taipei Medical University

[2] Department of Emergency and Critical Medicine, Wan Fang Hospital, Taipei Medical University, Taipei, Taiwan

[3] Department of Emergency Medicine, Tri-Service General Hospital, National Defense Medical Center, Taipei, Taiwan

[4] Institute of Clinical Medicine, National Yang-Ming University, Taipei, Taiwan

[5] Department of Medical Research, Tri-Service General Hospital, National Defense Medical Center

[6] School of Public Health, National Defense Medical Center, Taipei, Taiwan

[7] Taiwanese Injury Prevention and Safety Promotion Association, Taipei, Taiwan

[8] Department of Pediatrics, Taoyuan Armed Forces General Hospital, Taoyuan, Taiwan

[*] W.-C. Chien and S.-H. Tsai contributed equally to this work and are co-corresponding authors

*Corresponding authors:

Dr. Shih-Hung Tsai

Department of Emergency Medicine,

Tri-Service General Hospital, National Defense Medical Center, Taipei, Taiwan

No. 325, Sec. 2, Cheng-Kung Road, Neihu Dist., Taipei City 11490, Taiwan

Tel: + 886-2-87923311-16877;

Fax: + 886-2-87927034

E-mail: tsaishihung@yahoo.com.tw;

Dr. Wu-Chien Chien

Department of Medical Research 7115R,

Tri-Service General Hospital, National, Defense Medical Center, No.325, Section 2, Cheng-Kung Road, Neihu District, Taipei City, 11490, Taiwan

Tel: + 886-2-87923100

E-mail: chienwu@mail.ndmctsgh.edu.tw

For peer review only - http://bmjopen.bmj.com/site/about/guidelines.xhtml

Abstract

Objective Marfan syndrome (MFS) involves a deficiency of the structural extracellular matrix component fibrillin-1 (FBN-1) and over-activation of the transforming growth factor-β (TGF-β) signaling pathway. The TGF-β signaling pathway also actively participates in malignant transformation. Although anecdotal case reports have suggested associations between MFS/MFS-like conditions and several hematological and solid malignancies, such associations have not been thoroughly evaluated in large-scale studies. We sought to use a nationwide health care insurance claim database to evaluate whether patients with MFS are at increased risk of malignancy.

Patients and Methods We conducted a nested case-control analysis using a database extracted from Taiwan's National Health Insurance Research Database (NHIRD). All medical conditions for each case and control were categorized using ICD-9 classifications. Odds ratios (ORs) and 95% confidence intervals (CIs) for associations between MFS and malignancies were estimated using conditional logistic regression and adjusted for comorbidities.

Results Our analyses included 1,153,137 cancer cases and 1,153,137 propensity score-matched controls. Relative to other subjects, patients with MFS exhibited significantly higher rates of malignancy and hypertension and were significantly more

For peer review only

likely to be male. Malignancies originating from the head and neck and the urinary tract were significantly more frequent among patients with MFS than among subjects without MFS.

Conclusion Patients with MFS are at increased risk of developing various malignancies. Health care professionals should be aware of this risk when treating such patients, and increased cancer surveillance may be necessary for these patients.

Key words: Marfan syndrome, malignancy, transforming growth factor-β, fibrillin,

For peer review only

For peer review only - http://bmjopen.bmj.com/site/about/guidelines.xhtml

Strengths and limitations of this study

- The associations between MFS/MFS-like conditions and malignancies have not been thoroughly evaluated in large-scale studies. In this respect, we used a nationwide health care insurance claim database to evaluate whether patients with MFS are at increased risk of malignancy.
- Patients with MFS are at increased risk of developing various malignancies. Health care professionals should be aware of this risk when treating such patients.
- NHIRD registry could not provide detailed information regarding laboratory results, family histories and health-related lifestyle factors, that could increase the risk of malignancy and were potential confounding factors in this study.

For peer review only

For peer review only - http://bmjopen.bmj.com/site/about/guidelines.xhtml

Introduction

Marfan syndrome (MFS) is a pleiotropic connective tissue disease caused by a deficiency of the structural extracellular matrix component fibrillin-1 (FBN-1). The study of murine models of MFS has revealed the involvement of the transforming growth factor-β (TGF-β) signaling pathway in the pathogenesis of this disease. Overactivated TGF-β signaling is associated with MFS and several MFS-like conditions, including Loeys-Dietz syndrome, Shprintzen-Goldberg syndrome, aneurysm-osteoarthritis syndrome and syndromic thoracic aortic aneurysm. These conditions are also clearly associated with degenerative non-inflammatory structural cardiovascular diseases, including aortic root dilatation, thoracic aneurysm and aortic dissection.(1, 2) The TGF-β signaling pathway also actively participates in malignant transformation. In tumor cells, TGF-β loses its anti-proliferative response and becomes an oncogenic factor; as a result, TGF-β function is impaired in various solid and hematological malignancies.(3) TGF-β-induced epithelial-mesenchymal transition and reversion from mesenchymal to epithelial phenotypes contribute to the survival and dissemination of malignant cells.(4) Anecdotal case reports have suggested an association between MFS/MFS-like conditions and several hematological and solid malignancies.(5-15) However, associations between MFS/MFS-like conditions and malignancies have not been thoroughly evaluated in large-scale studies. In this respect,

For peer review only - http://bmjopen.bmj.com/site/about/guidelines.xhtml

we sought to use a nationwide health care insurance claim database to evaluate whether patients with MFS are at increased risk of malignancy.

For peer review only

For peer review only - http://bmjopen.bmj.com/site/about/guidelines.xhtml

Methods

Data source

Data for our nationwide, population-based, nested case-control study were obtained from inpatient care records and registration files from the Taiwan National Health Insurance Research Database (NHIRD). The National Health Insurance program was implemented in 1995 and provides health care coverage to 99% of the Taiwanese population (more than 23 million people). The accuracy of the NHIRD with respect to diagnoses for major diseases, such as stroke and acute coronary syndrome, has been validated.(16, 17) The confidentiality of individuals was protected by utilizing encrypted personal identification to avoid the possibility of ethical violations related to the study data. This investigation was conducted in accordance with the Declaration of Helsinki and other relevant guidelines. This study was approved by the Institutional Review Board of Tri-Service General Hospital, National Defense Medical Center, Taipei, Taiwan (TSGH IRB No. B-104-21).

Cancer cases and controls

This study involved a nested case-control design. Using the NHIRD, we selected adult patients>18 years of age who had been diagnosed with a malignancy based on ICD-9-DM codes (140-208) between 2000 and 2013 and confirmed these patients'

For peer review only - http://bmjopen.bmj.com/site/about/guidelines.xhtml

diagnoses by linking them to cases registered in the Catastrophic Illness Patient Database. The date of the first malignancy diagnosis was defined as the index date. We excluded patients who had been diagnosed with Marfan syndrome (ICD-9-CM 759.82) within 6 months prior to being diagnosed with a new malignancy. A sample of control candidates was selected for comparison from individuals in the NHIRD who were without malignancies. Patients in the study and control groups were selected via 1:1 matching by age, sex, and comorbidities, including hypertension (ICD-9-CM 401-405), diabetes (ICD-9-CM 250), hyperlipidemia (ICD-9-CM 272.0-272.4), chronic obstructive pulmonary disease (COPD) (ICD-9-CM 490-496), alcoholism (ICD-9-CM 303), and obesity (ICD-9-CM 278).

Outcome measurements

Only patients diagnosed with MFS prior to the index date were considered. MFS was identified from the NHIRD by utilizing the corresponding ICD-9 code (ICD-9-CM code 759.82). The following covariates were included: age, gender, hypertension, diabetes mellitus (DM), hyperlipidemia, COPD, alcoholism and obesity.

Statistical analysis

Patients' clinical characteristics were expressed in numerical form. Categorical variables, which were presented as percentages, were compared using chi-square tests

For peer review only - http://bmjopen.bmj.com/site/about/guidelines.xhtml

and Fisher's exact test. Continuous variables, which were presented as means and standard deviations, were compared using t-tests. The primary goal of this study was to determine whether a patient's clinical characteristics, such as MFS, were associated with malignancies. Associations between those outcomes (prognoses) and clinical characteristics were investigated using conditional logistic regression in a generalized estimating equation (GEE) model. The regression results are presented as adjusted odds ratios (ORs) with corresponding 95% confidence intervals (CIs). The threshold for statistical significance was $p<0.05$. All data analyses were conducted using SPSS version 18 (SPSS, Inc., Chicago, IL, USA).

For peer review only

For peer review only - http://bmjopen.bmj.com/site/about/guidelines.xhtml

Results

A flow diagram of the patient enrollment scheme is presented in **Figure 1**. During the study period, a total of 1,153,137 patients in follow-up after being diagnosed with a malignancy were identified in the NHIRD, which included a total of 13,139,306 inpatients. Other patients from the same database who did not have malignancies and were matched by age, gender, and comorbidities were designated as controls. As indicated in **Table 1**, as expected, there were no statistically significant differences between the case and control groups with respect to gender, age and co-morbidities, including hypertension, DM, hyperlipidemia, COPD, alcoholism and obesity, after matching. At the end of the 13-year study period, the incidence of MFS was significantly higher among patients with malignancies compared with patients without malignancies over the corresponding observation period (0.008% vs. 0.003%, p<0.001). **Table 2** indicates that malignancy (adjusted OR=3.991, 95% CI: 2.555-6.235, p<0.001), maleness (adjusted OR=2.103, 95% CI: 1.427-3.098, p<0.001) and hypertension (adjusted OR=1.964, 95% CI: 1.258-3.064, p=0.013) were significantly associated with patients with MFS.

As indicated in **Table 3**, among patients with MFS, malignancies originating from the head and neck (8.60% vs. 1.25%, p=0.013) and the urinary tract (11.83% vs. 5.61%, p=0.040) were significantly more frequent among patients with MFS than

For peer review only - http://bmjopen.bmj.com/site/about/guidelines.xhtml

among subjects without MFS. In contrast, there were significantly lower incidences of lung (6.45% vs. 12.56%, p=0.048) and breast (1.08% vs. 8.54%, p=0.029) malignancies among patients with MFS than among patients without MFS. There were no statistically significant differences between patients with and without MFS with respect to either malignancies of hematological origin or malignancies originating from the gastrointestinal tract, female genital tract, or thyroid.

For peer review only

For peer review only - http://bmjopen.bmj.com/site/about/guidelines.xhtml

Discussion

Using a nationwide population database, we have demonstrated for the first time that MFS is associated with the development of malignancies. No systemic approaches have previously been utilized to elucidate associations between MFS and malignancies.

Numerous case reports have addressed associations between MFS and various solid malignancies, including thyroid cancer,(8) osteosarcoma,(9) mesothelioma,(14) esophageal cancer,(18) angiosarcoma,(19) testicular cancer,(20) and Wilms' tumor.(21) Multiple endocrine neoplasia syndromes that are characterized by benign and malignant changes in multiple endocrine organs and incidental changes in nervous, muscular and connective tissues can also manifest marfanoid features.(22, 23) In our study, we observed that the frequencies of head and neck and urinary tract malignancies were significantly increased in patients with MFS. Accumulated case reports have also indicated that MFS and MFS-like conditions, such as Loeys-Dietz syndrome and Ehlers-Danlos syndrome, might be associated with various hematological malignancies and NHL.(5-7, 10-13, 15, 24), (25, 26) Larger, international studies with greater sample sizes should be conducted to study those associations.

For peer review only - http://bmjopen.bmj.com/site/about/guidelines.xhtml

For peer review only

As integral components of microfibrils, which provide strength and elasticity to the extracellular matrix, fibrillins, particularly FBN-1 and FBN-2, are thought to be involved in cancer pathogenesis and maintenance of the pluripotency of embryonic stem cells. Fibrillins are important for controlling the growth and differentiation of cells that they surround via interaction with integrins and growth factors as well as regulation of members of the TGF-β superfamily.(27) An FBN-1 deficiency impairs targeting of the large latent complex by the extracellular matrix, resulting in the unrestrained release of TGF-β ligands. An elevated TGF-β level in patients with MFS is correlated with larger aortic root diameters and faster aortic root growth.(28) TGF-β actively participates in malignant transformation and progression. Evidence has revealed associations between TGF-β and various solid malignancies, including head and neck cancers,(29) bladder cancer,(30) and prostate,(31) colon,(32) lung,(33) breast,(34) liver,(35) and renal cell carcinomas.(36) Mutations in TGFBR2, a putative tumor suppressor gene implicated in several malignancies, are also associated with inherited connective-tissue disorders.(37) The TGF-β/SMAD signaling pathway is constitutively activated in natural killer cells in acute lymphoblastic leukemia patients but not in healthy controls.(38) Functional variants of TGF-β1 genes may be significantly associated with the etiopathogenesis of acute myeloid leukemia.(39)

For peer review only

TGF-β1 also induces the PI3K/Akt/NF-κB signaling pathway during the recruitment of malignant cells in chronic myeloid leukemia.(40) A previous study performed in our laboratory has confirmed the association between malignancies and aortic aneurysms (unpublished data).

Limitations

The strength of our study is its population-based database design. We identified malignancies using the dual approaches of assessing ICD-9 code records and searching the registry of the Catastrophic Illness Patient Database to increase the accuracy of our data. We excluded confounding factors of malignancy, including comorbidities. Although we extensively adjusted our results by utilizing multivariate logistic regression models, our study nonetheless exhibited several limitations and did not address certain confounders. First, MFS might be underdiagnosed in Asian populations.(41) MFS is diagnosed using the Ghent criteria, which are primarily based on clinical features of Caucasian MFS populations; however, clinical features of the cardiovascular, ocular and skeletal systems significantly differ between Caucasian and Asian MFS populations.(41) Second, the NHIRD registry could not provide detailed information regarding laboratory results, family histories and health-related lifestyle factors, such as alcohol consumption or tobacco use, that can

For peer review only - http://bmjopen.bmj.com/site/about/guidelines.xhtml

increase the risk of malignancy and were potential confounding factors in this study. In our investigation, we also considered COPD incidence as a proxy variable for tobacco use to diminish the potential confounding effect of tobacco use on our results. (42) Third, this investigation was designed as a case-control study because this approach is efficient for relatively rare diseases or diseases with a long latency period, such as MFS, due to cost- and time-related considerations. Our study can identify associations between MFS and malignancies, but a case-control study cannot prove a cause-effect relationship. Further prospective follow-up studies should be considered.

Conclusion

Patients with MFS are at increased risk of developing various malignancies. Health care professionals should be aware of this risk when treating patients with MFS. Relative to other patients, patients with MFS may require additional cancer surveillance.

Author's contributors

C-WH and M-TL thought of the concept and designed the study. J-CW, W-IL and S-HT reviewed relevant literatures and drafted the manuscript. W-CC provided the dataset and coordinated the study. C-HC, C-HT and Y-FW analysed the data. All

For peer review only - http://bmjopen.bmj.com/site/about/guidelines.xhtml

authors collected and interpreted the data and approved the manuscript

Competing interests: none declared.

Data sharing statement: no additional data are available.

Acknowledgments

This study was supported by grants from Tri-Service General Hospital, National Defense Medical Center, Taipei, Taiwan (TSGH-C106-002; TSGH-C106-048); Taoyuan Armed Forces General Hospital, Taoyuan, Taiwan (10514); and the Ministry of Science and Technology (MOST-104-016 -043 -MY2).

For peer review only

Legends to tables and figures

Table 1 Baseline characteristics of the study population.

Table 2 Factors of Marfan syndrome determined using conditional logistic regression in a generalized estimating equation model.

Table 3 Comparisons of malignancies in patients with and without Marfan syndrome.

Figure 1 Patient selection flowchart.

For peer review only

For peer review only - http://bmjopen.bmj.com/site/about/guidelines.xhtml

References

1. Halushka MK, Angelini A, Bartoloni G, Basso C, Batoroeva L, Bruneval P, et al. Consensus statement on surgical pathology of the aorta from the Society for Cardiovascular Pathology and the Association For European Cardiovascular Pathology: II. Noninflammatory degenerative diseases - nomenclature and diagnostic criteria. Cardiovascular pathology : the official journal of the Society for Cardiovascular Pathology. 2016;25(3):247-57.

2. Cook JR, Carta L, Galatioto J, Ramirez F. Cardiovascular manifestations in Marfan syndrome and related diseases; multiple genes causing similar phenotypes. Clinical genetics. 2015;87(1):11-20.

3. Kubiczkova L, Sedlarikova L, Hajek R, Sevcikova S. TGF-beta - an excellent servant but a bad master. Journal of translational medicine. 2012;10:183.

4. Katsuno Y, Lamouille S, Derynck R. TGF-beta signaling and epithelial-mesenchymal transition in cancer progression. Current opinion in oncology. 2013;25(1):76-84.

5. Corso A, Pagnucco G, Morra E, Bernasconi C. [The diagnosis of non-Hodgkin's lymphoma in a patient with Marfan's syndrome]. Minerva medica. 1993;84(7-8):417-9.

6. Sharief N, Kingston JE, Wright VM, Costeloe K. Acute leukemia in an infant with Marfan's syndrome: a case report. Pediatric hematology and oncology. 1991;8(4):323-7.

7. Miyajima Y, Kitase Y, Mizuno S, Sakai H, Matsumoto N, Ogawa A. [Acute lymphoblastic leukemia in a pediatric patient with Marfan's syndrome]. [Rinsho ketsueki] The Japanese journal of clinical hematology. 2011;52(1):28-31.

8. Winer DA, Winer S, Rotstein L, Asa SL, Mete O. Villous papillary thyroid carcinoma: a variant associated with marfan syndrome. Endocrine pathology. 2012;23(4):254-9.

9. Roopnariane A, Freed RJ, Price S, Fox EJ, Ritty TM. Osteosarcoma in a Marfan patient with a novel premature termination codon in the FBN1 gene. Connective tissue research. 2011;52(2):157-65.

10. Yoshitake K, Hagiwara Y, Tanae K, Takahashi N, Kohri M, Tamaru J, et al. [Marfan syndrome complicated with CD5+ CD10+ diffuse large B-cell lymphoma]. [Rinsho ketsueki] The Japanese journal of clinical hematology. 2010;51(3):196-200.

11. Lachanas E, Tomos P, Fotinou M, Miyakis S. Lymphoma presenting as chronic aortic dissection in Marfan syndrome. Annals of hematology. 2006;85(3):181-2.

12. Kraemer DM, Waschke J, Kunzmann V, Wilhelm M. Veno-occlusive disease in a male patient with Marfan syndrome and common acute lymphoblastic leukemia

For peer review only - http://bmjopen.bmj.com/site/about/guidelines.xhtml

during induction therapy. Annals of hematology. 2003;82(7):444-7.
13. Lee JJ, Kim HJ, Chung IJ, Jeong MH, Kook H, Seo JS, et al. A case of Marfan syndrome with acute monoblastic leukemia. The Korean journal of internal medicine. 1998;13(2):140-2.
14. Bisconti M, Bisetti A, Bidoli P. Malignant mesothelioma in subjects with Marfan's syndrome and Ehlers-Danlos syndrome: only an apparent association? Respiration; international review of thoracic diseases. 2000;67(2):223-8.
15. Togashi Y, Sakoda H, Sugahara H, Asagoe K, Matsuzawa Y. [Loeys-Dietz syndrome with acute myeloid leukemia]. [Rinsho ketsueki] The Japanese journal of clinical hematology. 2008;49(8):664-7.
16. Cheng CL, Kao YH, Lin SJ, Lee CH, Lai ML. Validation of the National Health Insurance Research Database with ischemic stroke cases in Taiwan. Pharmacoepidemiol Drug Saf. 2011;20(3):236-42.
17. Mao CT, Tsai ML, Wang CY, Wen MS, Hsieh IC, Hung MJ, et al. Outcomes and characteristics of patients undergoing percutaneous angioplasty followed by below-knee or above-knee amputation for peripheral artery disease. PloS one. 2014;9(10):e111130.
18. Milleron O, Arnoult F, Ropers J, Aegerter P, Detaint D, Delorme G, et al. Marfan Sartan: a randomized, double-blind, placebo-controlled trial. European heart journal. 2015;36(32):2160-6.
19. Okada M, Takeuchi E, Mori Y, Ichihara S, Usui A, Ueda Y. An autopsy case of angiosarcoma arising around a woven Dacron prosthesis after a Cabrol operation. The Journal of thoracic and cardiovascular surgery. 2004;127(6):1843-5.
20. Dexeus FH, Logothetis CJ, Chong C, Sella A, Ogden S. Genetic abnormalities in men with germ cell tumors. The Journal of urology. 1988;140(1):80-4.
21. Newbold SG, Shafer AD, Goodwin CD, Nanagas VN, Dimlich SH. Stage III Wilms' tumor of a solitary kidney in a patient with Marfan's syndrome: a 5-yr survival. Journal of pediatric surgery. 1982;17(6):841-2.
22. Fassbender WJ, Krohn-Grimberghe B, Gortz B, Litzlbauer D, Stracke H, Raue F, et al. Multiple endocrine neoplasia (MEN)--an overview and case report--patient with sporadic bilateral pheochromocytoma, hyperparathyroidism and marfanoid habitus. Anticancer research. 2000;20(6c):4877-87.
23. Sotos JG. Abraham Lincoln's marfanoid mother: the earliest known case of multiple endocrine neoplasia type 2B? Clinical dysmorphology. 2012;21(3):131-6.
24. Papadopoulos A, Ntaios G, Kaiafa G, Girtovitis F, Saouli Z, Kontoninas Z, et al. Veno-occlusive disease of the liver during induction therapy for acute lymphoblastic leukemia. International journal of hematology. 2008;88(4):441-2.
25. Bell D, Marshman D. Diffuse Large B Cell Lymphoma in a Prosthetic Aortic Graft.

For peer review only - http://bmjopen.bmj.com/site/about/guidelines.xhtml

Heart, lung & circulation. 2017;26(2):e4-e6.

26. Ghosh S, Ghosh S, Kumar Ghosh S. Aortic aneurysm and non-Hodgkin's lymphoma in Marfan syndrome. Turkish journal of haematology : official journal of Turkish Society of Haematology. 2009;26(1):31-3.

27. Davis MR, Andersson R, Severin J, de Hoon M, Bertin N, Baillie JK, et al. Transcriptional profiling of the human fibrillin/LTBP gene family, key regulators of mesenchymal cell functions. Molecular genetics and metabolism. 2014;112(1):73-83.

28. Franken R, den Hartog AW, de Waard V, Engele L, Radonic T, Lutter R, et al. Circulating transforming growth factor-beta as a prognostic biomarker in Marfan syndrome. International journal of cardiology. 2013;168(3):2441-6.

29. Liu JF, Mao L, Bu LL, Ma SR, Huang CF, Zhang WF, et al. C4.4A as a biomarker of head and neck squamous cell carcinoma and correlated with epithelial mesenchymal transition. American journal of cancer research. 2015;5(12):3505-15.

30. Liang Y, Zhu F, Zhang H, Chen D, Zhang X, Gao Q, et al. Conditional ablation of TGF-beta signaling inhibits tumor progression and invasion in an induced mouse bladder cancer model. Scientific reports. 2016;6:29479.

31. Korrodi-Gregorio L, Silva JV, Santos-Sousa L, Freitas MJ, Felgueiras J, Fardilha M. TGF-beta cascade regulation by PPP1 and its interactors -impact on prostate cancer development and therapy. Journal of cellular and molecular medicine. 2014;18(4):555-67.

32. Ramamoorthi G, Sivalingam N. Molecular mechanism of TGF-beta signaling pathway in colon carcinogenesis and status of curcumin as chemopreventive strategy. Tumour biology : the journal of the International Society for Oncodevelopmental Biology and Medicine. 2014;35(8):7295-305.

33. Jeon HS, Jen J. TGF-beta signaling and the role of inhibitory Smads in non-small cell lung cancer. Journal of thoracic oncology : official publication of the International Association for the Study of Lung Cancer. 2010;5(4):417-9.

34. Zarzynska JM. Two faces of TGF-beta1 in breast cancer. Mediators of inflammation. 2014;2014:141747.

35. Yoshida K, Murata M, Yamaguchi T, Matsuzaki K. TGF-beta/Smad signaling during hepatic fibro-carcinogenesis (review). International journal of oncology. 2014;45(4):1363-71.

36. Bostrom AK, Lindgren D, Johansson ME, Axelson H. Effects of TGF-beta signaling in clear cell renal cell carcinoma cells. Biochem Biophys Res Commun. 2013;435(1):126-33.

37. Mizuguchi T, Collod-Beroud G, Akiyama T, Abifadel M, Harada N, Morisaki T, et al. Heterozygous TGFBR2 mutations in Marfan syndrome. Nature genetics. 2004;36(8):855-60.

For peer review only - http://bmjopen.bmj.com/site/about/guidelines.xhtml

38. Rouce RH, Shaim H, Sekine T, Weber G, Ballard B, Ku S, et al. The TGF-beta/SMAD pathway is an important mechanism for NK cell immune evasion in childhood B-acute lymphoblastic leukemia. Leukemia. 2016;30(4):800-11.

39. Nursal AF, Pehlivan M, Sahin HH, Pehlivan S. The Associations of IL-6, IFN-gamma, TNF-alpha, IL-10, and TGF-beta1 Functional Variants with Acute Myeloid Leukemia in Turkish Patients. Genetic testing and molecular biomarkers. 2016;20(9):544-51.

40. Zhu B, Zhang J, Chen J, Li C, Wang X. Molecular biological characteristics of the recruitment of hematopoietic stem cells from bone marrow niche in chronic myeloid leukemia. International journal of clinical and experimental pathology. 2015;8(10):12595-607.

41. Franken R, den Hartog AW, van de Riet L, Timmermans J, Scholte AJ, van den Berg MP, et al. Clinical features differ substantially between Caucasian and Asian populations of Marfan syndrome. Circulation journal : official journal of the Japanese Circulation Society. 2013;77(11):2793-8.

42. Yu TM, Chuang YW, Yu MC, Chen CH, Yang CK, Huang ST, et al. Risk of cancer in patients with polycystic kidney disease: a propensity-score matched analysis of a nationwide, population-based cohort study. The Lancet Oncology. 2016.

For peer review only

Table 1 Baseline characteristics of the study population.

	Patients with malignancy N=1,153,137	Patients without malignancy N=1,153,137	*P* value
Marfan syndrome	93 (0.008%)	31 (0.003%)	<0.001
Gender			0.999
Male	657,188 (56.990%)	657,188 (56.990%)	
Female	495,969 (43.010%)	495,969 (43.010%)	
Age (years)	63.52±15.21	62.63±15.93	0.724
Comorbidities			
Hypertension	150,555 (13.056%)	150,462 (13.048%)	0.945
Diabetes	127,884 (11.090%)	127,813 (11.085%)	0.980
Hyperlipidemia	7,235 (0.627%)	7,239 (0.628%)	0.996
COPD	48,668 (4.220%)	48,607 (4.215%)	0.963
Alcoholism	1,052 (0.091%)	1,124 (0.097%)	0.911
Obesity	230 (0.020%)	294 (0.025%)	0.928

COPD=chronic obstructive pulmonary disease;

For peer review only

Table 2 Factors of Marfan syndrome determined using conditional logistic regression in a generalized estimating equation model.

	Crude OR*	95%CI	P value	Adjust OR	95%CI	P value
Malignancy						
Without	Reference			Reference		
With	3.000	1.998-4.505	<0.001	3.991	2.555-6.236	<0.001
Gender						
Male	2.183	1.486-3.207	<0.001	2.103	1.427-3.098	<0.001
Female	Reference			Reference		
Hypertension						
Without	Reference			Reference		
With	1.529	1.014-2.306	0.003	1.964	1.258-3.064	0.013
Diabetes						
Without	Reference			Reference		
With	0.992	0.587-1.677	0.976	0.960	0.555-1.659	0.882
Hyperlipidemia						
Without	Reference			Reference		
With	0.739	0.183-2.989	0.672	0.864	0.209-3.571	0.840
COPD						
Without	Reference			Reference		
With	0.168	0.024-1.205	0.076	0.169	0.024-1.221	0.078
Alcoholism						
Without	Reference			Reference		
With	0.000	-	0.985	0.000	-	0.987
Obesity						
Without	Reference			Reference		
With	1.009	0.888-1.017	0.138	1.006	0.816-1.012	0.253

*OR= odds ratio, CI = confidence interval, Adjusted OR: Adjusted variables listed in the table

For peer review only

Table 3 Comparisons of malignancies in patients with and without Marfan syndrome.

	Marfan syndrome		Control group		P value
	N	%	N	%	
Cancers	93	0.01	1,153,044	99.99	
Head and neck	8	8.60	14,394	1.25	0.013
Esophagus	6	6.45	26,321	2.28	0.288
Stomach	6	6.45	57,991	5.03	0.827
Colon and rectum	9	9.68	136,480	11.84	0.735
Liver	11	11.83	172,837	14.99	0.532
Trachea, bronchus and lung	6	6.45	144,792	12.56	0.048
Female breast	1	1.08	98,414	8.54	0.029
Female genital organs	5	5.38	68,716	5.96	0.956
Prostate	7	7.53	47,019	4.08	0.578
Bladder , ureter and kidney	11	11.83	64,648	5.61	0.040
Thyroid	6	6.45	23,584	2.05	0.312
Hematopoietic malignancy	7	7.53	53,206	4.61	0.711
Others	10	10.75	244,642	21.22	0.003

For peer review only - http://bmjopen.bmj.com/site/about/guidelines.xhtml

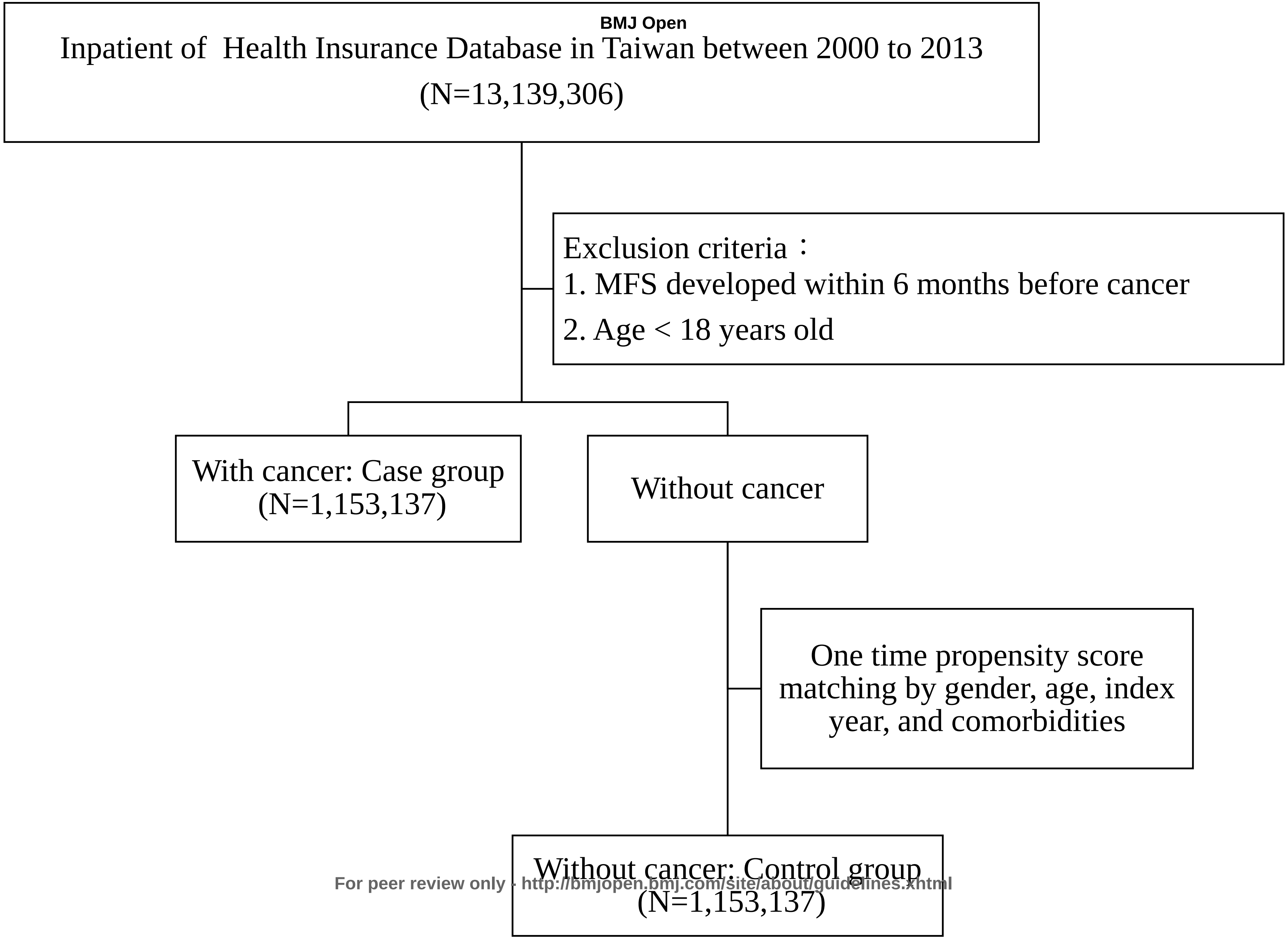

For peer review only - http://bmjopen.bmj.com/site/about/guidelines.xhtml

STROBE 2007 (v4) Statement—Checklist of items that should be included in reports of *case-control studies*

Section/Topic	Item #	Recommendation	Reported on page #
**Title and abstract**	1	(*a*) Indicate the study's design with a commonly used term in the title or the abstract	1
		(*b*) Provide in the abstract an informative and balanced summary of what was done and what was found	2-3
**Introduction**			
Background/rationale	2	Explain the scientific background and rationale for the investigation being reported	4-5
Objectives	3	State specific objectives, including any prespecified hypotheses	6
**Methods**			
Study design	4	Present key elements of study design early in the paper	
Setting	5	Describe the setting, locations, and relevant dates, including periods of recruitment, exposure, follow-up, and data collection	6
Participants	6	(*a*) Give the eligibility criteria, and the sources and methods of case ascertainment and control selection. Give the rationale for the choice of cases and controls	7
		(*b*) For matched studies, give matching criteria and the number of controls per case	7-8
Variables	7	Clearly define all outcomes, exposures, predictors, potential confounders, and effect modifiers. Give diagnostic criteria, if applicable	8
Data sources/ measurement	8*	For each variable of interest, give sources of data and details of methods of assessment (measurement). Describe comparability of assessment methods if there is more than one group	
Bias	9	Describe any efforts to address potential sources of bias	
Study size	10	Explain how the study size was arrived at	10
Quantitative variables	11	Explain how quantitative variables were handled in the analyses. If applicable, describe which groupings were chosen and why	8-9
Statistical methods	12	(*a*) Describe all statistical methods, including those used to control for confounding	8-9
		(*b*) Describe any methods used to examine subgroups and interactions	n/a
		(*c*) Explain how missing data were addressed	n/a
		(*d*) If applicable, explain how matching of cases and controls was addressed	8
		(*e*) Describe any sensitivity analyses	n/a
**Results**			

For peer review only

Participants	13*	(a) Report numbers of individuals at each stage of study—eg numbers potentially eligible, examined for eligibility, confirmed eligible, included in the study, completing follow-up, and analysed	
		(b) Give reasons for non-participation at each stage	10
		(c) Consider use of a flow diagram	10
Descriptive data	14*	(a) Give characteristics of study participants (eg demographic, clinical, social) and information on exposures and potential confounders	10
		(b) Indicate number of participants with missing data for each variable of interest	n/a
Outcome data	15*	Report numbers in each exposure category, or summary measures of exposure	10
Main results	16	(*a*) Give unadjusted estimates and, if applicable, confounder-adjusted estimates and their precision (eg, 95% confidence interval). Make clear which confounders were adjusted for and why they were included	10
		(*b*) Report category boundaries when continuous variables were categorized	10
		(*c*) If relevant, consider translating estimates of relative risk into absolute risk for a meaningful time period	n/a
Other analyses	17	Report other analyses done—eg analyses of subgroups and interactions, and sensitivity analyses	n/a
**Discussion**			
Key results	18	Summarise key results with reference to study objectives	12
Limitations	19	Discuss limitations of the study, taking into account sources of potential bias or imprecision. Discuss both direction and magnitude of any potential bias	14
Interpretation	20	Give a cautious overall interpretation of results considering objectives, limitations, multiplicity of analyses, results from similar studies, and other relevant evidence	14
Generalisability	21	Discuss the generalisability (external validity) of the study results	15
**Other information**			
Funding	22	Give the source of funding and the role of the funders for the present study and, if applicable, for the original study on which the present article is based	15

*Give information separately for cases and controls in case-control studies and, if applicable, for exposed and unexposed groups in cohort and cross-sectional studies.

Note: An Explanation and Elaboration article discusses each checklist item and gives methodological background and published examples of transparent reporting. The STROBE checklist is best used in conjunction with this article (freely available on the Web sites of PLoS Medicine at http://www.plosmedicine.org/, Annals of Internal Medicine at http://www.annals.org/, and Epidemiology at http://www.epidem.com/). Information on the STROBE Initiative is available at www.strobe-statement.org.

For peer review only

BMJ Open

The association between malignancies and Marfan syndrome: a population-based, nested case-control study in Taiwan

Journal:	*BMJ Open*
Manuscript ID	bmjopen-2017-017243.R1
Article Type:	Research
Date Submitted by the Author:	11-Aug-2017
Complete List of Authors:	Hsu, Chin-Wang ; School of Medicine, College of Medicine, Taipei Medical University, Department of Emergency Medicine,<br>Wang, Jen-Chun; Tri-Service General Hospital, National Defensive Medical Center, department of Emergency Medicine<br>Liao, Wen-I; Emergency Medicine<br>Chien, Wu-Chien ; National Defense Medical Center<br>Chung, Chi-Hsiang; National Defense Medical Center<br>Tsao, Chang-Huei ; Tri-Service General Hospital, National Defensive Medical Center, department of Emergency Medicine, Department of Medical Research<br>Wu , Yung-Fu ; Tri-Service General Hospital, National Defense Medical Center<br>Liao, Min-Tser ; Taoyuan Armed Forces General Hospital,<br>Tsai, Shih-Hung; Tri-Service General Hospital, National Defense Medical Center, Department of Emeregncy Medicine
**Primary Subject Heading**:	Oncology
Secondary Subject Heading:	Genetics and genomics, Oncology
Keywords:	Marfan syndrome, Malignancy, fibrillin, transforming growth factor-β

SCHOLARONE™ Manuscripts

The association between malignancies and Marfan syndrome: a population-based, nested case-control study in Taiwan

Chin-Wang Hsu, MD [1,2], Jen-Chun Wang, MD [3,4], Wen-I Liao, MD [3], Wu-Chien Chien, PhD [5,6,*], Chi-Hsiang Chung, PhD [5,6,7], Chang-Huei Tsao, PhD [5], Yung-Fu Wu, PhD [5], Min-Tser Liao, MD[8], Shih-Hung Tsai, MD, PhD [3,*]

[1] Department of Emergency Medicine, School of Medicine, College of Medicine, Taipei Medical University, Taipei, Taiwan

[2] Department of Emergency and Critical Medicine, Wan Fang Hospital, Taipei Medical University, Taipei, Taiwan

[3] Department of Emergency Medicine, Tri-Service General Hospital, National Defense Medical Center, Taipei, Taiwan

[4] Institute of Clinical Medicine, National Yang-Ming University, Taipei, Taiwan

[5] Department of Medical Research, Tri-Service General Hospital, National Defense Medical Center, Taipei, Taiwan

[6] School of Public Health, National Defense Medical Center, Taipei, Taiwan

[7] Taiwanese Injury Prevention and Safety Promotion Association, Taipei, Taiwan

[8] Department of Pediatrics, Taoyuan Armed Forces General Hospital, Taoyuan, Taiwan

[*] W.-C. Chien and S.-H. Tsai contributed equally to this work and are co-corresponding authors.

*Corresponding authors:

Dr. Shih-Hung Tsai

Department of Emergency Medicine,

Tri-Service General Hospital, National Defense Medical Center, Taipei, Taiwan

No. 325, Sec. 2, Cheng-Kung Road, Neihu Dist., Taipei City 11490, Taiwan

Tel.: + 886-2-87923311-16877

Fax: + 886-2-87927034

E-mail: tsaishihung@yahoo.com.tw

Dr. Wu-Chien Chien

Department of Medical Research 7115R,

Tri-Service General Hospital, National, Defense Medical Center, No. 325, Section 2, Cheng-Kung Road, Neihu District, Taipei City, 11490, Taiwan

Tel.: + 886-2-87923100

E-mail: chienwu@mail.ndmctsgh.edu.tw

For peer review only - http://bmjopen.bmj.com/site/about/guidelines.xhtml

Abstract

Objective Marfan syndrome (MFS) involves a deficiency of the structural extracellular matrix component fibrillin-1 (FBN-1) and over-activation of the transforming growth factor-β (TGF-β) signaling pathway. The TGF-β signaling pathway also actively participates in malignant transformation. Although anecdotal case reports have suggested associations between MFS/MFS-like conditions and several hematological and solid malignancies, such associations have not been thoroughly evaluated in large-scale studies. We sought to use a nationwide health care insurance claim database to evaluate whether patients with MFS are at increased risk of malignancy.

Patients and Methods We conducted a nested case-control analysis using a database extracted from Taiwan's National Health Insurance Research Database (NHIRD). All medical conditions for each case and control were categorized using ICD-9 classifications. Odds ratios (ORs) and 95% confidence intervals (CIs) for associations between MFS and malignancies were estimated using conditional logistic regression and adjusted for comorbidities.

Results Our analyses included 1,153,137 cancer cases and 1,153,137 propensity score-matched controls. Relative to other subjects, patients with MFS had a significantly higher risk of having a malignancy (adjusted odds ratio (OR): 3.991) and

For peer review only - http://bmjopen.bmj.com/site/about/guidelines.xhtml

hypertension (adjusted OR: 1.964) and were significantly more likely to be male. Malignancies originating from the head and neck and the urinary tract were significantly more frequent among patients with MFS than among subjects without MFS.

Conclusion Patients with MFS are at increased risk of developing various malignancies. Health care professionals should be aware of this risk when treating such patients, and increased cancer surveillance may be necessary for these patients.

Key words: Marfan syndrome, malignancy, transforming growth factor-β, fibrillin

For peer review only

Strengths and limitations of this study

- The associations between MFS/MFS-like conditions and malignancies have not been thoroughly evaluated in large-scale studies. Thus, we used a nationwide healthcare insurance claim database to evaluate whether patients with MFS are at increased risk of malignancy. We found that patients with MFS are at increased risk of developing various malignancies.
- The NHIRD registry did not provide detailed information regarding laboratory results, family history and health-related lifestyle factors that could increase the risk of malignancy, and these factors represent potential confounding factors in this study.

For peer review only - http://bmjopen.bmj.com/site/about/guidelines.xhtml

Introduction

Marfan syndrome (MFS) is a pleiotropic connective tissue disease caused by a deficiency of the structural extracellular matrix component fibrillin-1 (FBN-1). The study of murine models of MFS has revealed the involvement of the transforming growth factor-β (TGF-β) signaling pathway in the pathogenesis of this disease. Overactivated TGF-β signaling is associated with MFS and several MFS-like conditions, including Loeys-Dietz syndrome, Shprintzen-Goldberg syndrome, aneurysm-osteoarthritis syndrome and syndromic thoracic aortic aneurysm. These conditions are also clearly associated with degenerative non-inflammatory structural cardiovascular diseases, including aortic root dilatation, thoracic aneurysm and aortic dissection.(1, 2) The TGF-β signaling pathway also actively participates in malignant transformation. In tumor cells, TGF-β loses its anti-proliferative response and becomes an oncogenic factor; as a result, TGF-β function is impaired in various solid and hematological malignancies.(3) TGF-β-induced epithelial-mesenchymal transition and reversion from mesenchymal to epithelial phenotypes contribute to the survival and dissemination of malignant cells.(4) Anecdotal case reports have suggested an association between MFS/MFS-like conditions and several hematological and solid malignancies.(5-15) However, associations between MFS/MFS-like conditions and malignancies have not been thoroughly evaluated in large-scale studies. In this respect,

For peer review only - http://bmjopen.bmj.com/site/about/guidelines.xhtml

we sought to use a nationwide health care insurance claim database to evaluate whether patients with MFS are at increased risk of malignancy.

For peer review only

For peer review only - http://bmjopen.bmj.com/site/about/guidelines.xhtml

Methods

Data source

Data for our nationwide, population-based, nested case-control study were obtained from inpatient care records and registration files from the Taiwan National Health Insurance Research Database (NHIRD). The National Health Insurance program was implemented in 1995 and provides health care coverage to 99% of the Taiwanese population (more than 23 million people). The accuracy of the NHIRD with respect to diagnoses for major diseases, such as stroke and acute coronary syndrome, has been validated.(16, 17) The confidentiality of individuals was protected by utilizing encrypted personal identification to avoid the possibility of ethical violations related to the study data. This investigation was conducted in accordance with the Declaration of Helsinki and other relevant guidelines. This study was approved by the Institutional Review Board of Tri-Service General Hospital, National Defense Medical Center, Taipei, Taiwan (TSGH IRB No. B-104-21).

Cancer cases and controls

This study involved a nested case-control design. Using the NHIRD, we selected adult patients >18 years of age who had been diagnosed with a malignancy based on ICD-9-DM codes (140-208) between 2000 and 2013 and confirmed these patients'

For peer review only - http://bmjopen.bmj.com/site/about/guidelines.xhtml

diagnoses by linking them to cases registered in the Catastrophic Illness Patient Database. The date of the first malignancy diagnosis was defined as the index date. We identified patients with MFS using the ICD-9-CM code 759.82. A sample of control candidates was selected for comparison from individuals in the NHIRD who were without malignancies. Patients in the study and control groups were selected via 1:1 matching by age, sex, number of medical follow-ups and comorbidities, including hypertension (ICD-9-CM 401-405), diabetes (ICD-9-CM 250), hyperlipidemia (ICD-9-CM 272.0-272.4), chronic obstructive pulmonary disease (COPD) (ICD-9-CM 490-496), alcoholism (ICD-9-CM 303), and obesity (ICD-9-CM 278). Data on the use of angiotensin-converting enzyme inhibitors and angiotensin receptor blockers were acquired from the Longitudinal Health Insurance Database 2005 (LHID 2005), a sub-database of the NHIRD. All insurance claims were scrutinized by medical reimbursement specialists, and peer review was undertaken according to standard and clinical diagnostic criteria, such as the pulmonary function test for COPD. Therefore, the diagnoses of COPD in this study should be highly reliable. Both the NHIRD and catastrophic illness certificate have been well-validated internally and externally in several studies.(16, 18, 19)

Outcome measurements

Only patients diagnosed with MFS prior to the index date were considered. MFS

For peer review only - http://bmjopen.bmj.com/site/about/guidelines.xhtml

was identified from the NHIRD by utilizing the corresponding ICD-9 code (ICD-9-CM code 759.82). The following covariates were included: age, gender, hypertension, diabetes mellitus (DM), hyperlipidemia, COPD, alcoholism and obesity.

Statistical analysis

Patients' clinical characteristics were expressed in numerical form. Categorical variables, which were presented as percentages, were compared using chi-square tests and Fisher's exact test. Continuous variables, which were presented as the mean and standard deviation, were compared using t-tests. The primary goal of this study was to determine whether a patient's clinical characteristics, such as MFS, were associated with malignancies. Associations between those outcomes (prognoses) and clinical characteristics were investigated using conditional logistic regression in a generalized estimating equation (GEE) model. The regression results are presented as adjusted odds ratios (ORs) with corresponding 95% confidence intervals (CIs). The threshold for statistical significance was $p<0.05$. All data analyses were conducted using SPSS version 18 (SPSS, Inc., Chicago, IL, USA).

For peer review only - http://bmjopen.bmj.com/site/about/guidelines.xhtml

Results

A flow diagram of the patient enrollment scheme is presented in **Figure 1**. During the study period, a total of 1,153,137 patients in follow-up after being diagnosed with a malignancy were identified in the NHIRD, which included a total of 13,139,306 inpatients. Other patients from the same database who did not have malignancies and were matched by age, gender, and comorbidities were designated controls. As indicated in **Table 1**, as expected, there were no statistically significant differences between the case and control groups with respect to gender, age, number of medical follow-ups and comorbidities, including hypertension, DM, hyperlipidemia, COPD, alcoholism, and obesity, after matching.

Table 1 Baseline characteristics of the study population.

	Patients with malignancy N=1,153,137	Patients without malignancy N=1,153,137	*P* value
Marfan syndrome	93 (0.008%)	27 (0.002%)	<0.001
Gender			0.999
Male	657,188 (56.990%)	657,188 (56.990%)	
Female	495,969 (43.010%)	495,969 (43.010%)	
Age (years)	63.52±15.21	63.49±18.42	0.178
Comorbidities			
Hypertension	150,555 (13.056%)	150,512 (13.052%)	0.933
Diabetes	127,884 (11.090%)	127,894 (11.091%)	0.978
Hyperlipidemia	7,235 (0.627%)	7,233 (0.627%)	0.987
COPD	48,668 (4.220%)	48,656 (4.219%)	0.969
Alcoholism	1,052 (0.091%)	1,042 (0.090%)	0.827
Obesity	230 (0.020%)	247 (0.021%)	0.436

For peer review only - http://bmjopen.bmj.com/site/about/guidelines.xhtml

Medical follow-ups	4.84±10.70	4.82±10.87	0.159

COPD=chronic obstructive pulmonary disease.

At the end of the 13-year study period, the incidence of MFS was significantly higher among patients with malignancies compared with patients without malignancies over the corresponding observation period (0.008% vs. 0.003%, p<0.001). Among patients with MFS, there were no statistically significant differences regarding the use of angiotensin-converting enzyme inhibitors and angiotensin receptor blockers between patients with or without malignancies (20.93% vs 21.33%, p=0.95; 18.60% vs 19.11%, p=0.93, respectively). **Table 2** indicates that malignancy (adjusted OR=3.991, 95% CI: 2.555-6.235, p<0.001), maleness (adjusted OR=2.103, 95% CI: 1.427-3.098, p<0.001) and hypertension (adjusted OR=1.964, 95% CI: 1.258-3.064, p=0.013) were significantly associated with patients with MFS.

Table 2 Factors of Marfan syndrome determined using conditional logistic regression in a generalized estimating equation model.

	Crude OR*	95% CI	P value	Adjusted OR	95% CI	P value
Malignancy						
Without	Reference			Reference		
With	3.444	2.012-4.671	<0.001	3.999	2.578-6.301	<0.001
Gender						
Male	2.187	1.490-3.211	<0.001	2.110	1.429-3.101	<0.001
Female	Reference			Reference		
Hypertension						

For peer review only - http://bmjopen.bmj.com/site/about/guidelines.xhtml

Without	Reference			Reference		
With	1.503	1.013-2.324	0.001	1.962	1.224-3.000	0.010
Diabetes						
Without	Reference			Reference		
With	0.993	0.546-1.717	0.785	0.963	0.527-1.688	0.809
Hyperlipidemia						
Without	Reference			Reference		
With	0.748	0.197-2.862	0.704	0.711	0.214-3.201	0.764
COPD						
Without	Reference			Reference		
With	0.299	0.025-1.374	0.102	0.398	0.027-1.401	0.083
Alcoholism						
Without	Reference			Reference		
With	0.000	-	0.989	0.000	-	0.990
Obesity						
Without	Reference			Reference		
With	1.014	0.859-1.086	0.177	1.010	0.803-1.020	0.243

*OR=odds ratio; CI=confidence interval; Adjusted OR: adjusted by variables listed in the table.

As indicated in **Table 3**, malignancies originating from the head and neck (adjusted OR=8.714, 95% CI: 2.477-30.649, p<0.001), esophagus (adjusted OR=0.970, 95% CI: 3.943-30.522, p<0.001), stomach (adjusted OR=4.639, 95% CI: 1.743-12.345, p=0.002), colon and rectum (adjusted OR=3.134, 95% CI: 1.370-7.169, p=0.007), liver (adjusted OR=3.049, 95% CI: 1.349-6.980, p=0.007), female genital organs (adjusted OR=4.055, 95% CI: 1.417-11.606, p=0.009), prostate (adjusted OR=6.423, 95% CI: 2.355-17.517, p<0.001), urinary tract (adjusted OR=6.478, 95% CI: 2.974-14.112, p<0.001) and thyroid (adjusted OR=10.485, 95% CI: 4.251-25.861, p<0.001) and hematological malignancies (adjusted OR=6.981, 95% CI: 2.800-17.404,

For peer review only

For peer review only - http://bmjopen.bmj.com/site/about/guidelines.xhtml

p<0.001) were significantly more frequent among patients with MFS than among subjects without MFS.

Table 3 Analysis of malignancy subgroup using conditional logistic regression in a generalized estimating equation model in patients with or without Marfan syndrome.

Malignancy (with *vs.* without)	Adjusted OR	95% CI	P value
Total	3.999	2.578-6.301	<0.001
Head and neck	8.714	2.477-30.649	<0.001
Esophagus	10.970	3.943-30.522	<0.001
Stomach	4.639	1.743-12.345	0.002
Colon and rectum	3.134	1.370-7.169	0.007
Liver	3.049	1.349-6.980	0.007
Trachea, bronchus and lung	1.938	0.705-5.329	0.200
Female breast	0.477	0.060-3.782	0.483
Female genital organs	4.055	1.417-11.606	0.009
Prostate	6.423	2.355-17.517	<0.001
Bladder, ureter and kidney	6.478	2.974-14.112	<0.001
Thyroid	10.485	4.251-25.861	<0.001
Hematopoietic malignancy	6.981	2.800-17.404	<0.001
Others	2.900	1.434-5.863	0.003

Adjusted OR (odds ratio): adjusted for variables listed in Table 2; CI=confidence interval.

As indicated in **Table 4**, patients with MFS acquired malignancies at a younger age than those without MFS. During the study period, patients with MFS had a higher mortality rate and died younger than those without MFS..

Table 4 Analysis of age and survival regarding malignancy in patients with or without Marfan syndrome.

For peer review only - http://bmjopen.bmj.com/site/about/guidelines.xhtml

Marfan syndrome	With %	Without %	P value
Age at first inpatient visit due to malignancy	52.74±16.92	58.52±15.29	<0.001
Age at death during the follow-up period	67.35±1.63	70.84±11.85	<0.001
Patient mortality during the follow-up period	2.15%	0.197%	<0.001

* $p<0.05$

For peer review only

Discussion

Using a nationwide population database, we have demonstrated for the first time that MFS is associated with the development of certain malignancies. No systemic approaches have previously been utilized to elucidate associations between MFS and malignancies.

Numerous case reports have addressed associations between MFS and various solid malignancies, including thyroid cancer,(8) osteosarcoma,(9) mesothelioma,(14) esophageal cancer,(20) angiosarcoma,(21) testicular cancer,(22) and Wilms' tumor.(23) Multiple endocrine neoplasia syndromes that are characterized by benign and malignant changes in multiple endocrine organs and incidental changes in nervous, muscular and connective tissues can also manifest marfanoid features.(24, 25) In our study, we observed that the frequencies of head and neck and urinary tract malignancies were significantly increased in patients with MFS. Accumulated case reports have also indicated that MFS and MFS-like conditions, such as Loeys-Dietz syndrome and Ehlers-Danlos syndrome (EDS), might be associated with various hematological malignancies and NHL.(5-7, 10-13, 15, 26)(27, 28) Larger, international studies should be conducted to study these potential associations.

For peer review only - http://bmjopen.bmj.com/site/about/guidelines.xhtml

As integral components of microfibrils, which provide strength and elasticity to the extracellular matrix, fibrillins, particularly FBN-1 and FBN-2, are thought to be involved in cancer pathogenesis and maintenance of the pluripotency of embryonic stem cells. Fibrillins are important for controlling the growth and differentiation of cells that they surround via interaction with integrins and growth factors as well as regulation of members of the TGF-β superfamily.(29) An FBN-1 deficiency impairs targeting of the large latent complex by the extracellular matrix, resulting in the unrestrained release of TGF-β ligands. An elevated TGF-β level in patients with MFS is correlated with larger aortic root diameters and faster aortic root growth.(30) Dysregulated TGF-β2 is also associated with vascular EDS.(31) TGF-β actively participates in malignant transformation and progression. Evidence has revealed associations between TGF-β and various solid malignancies, including head and neck,(32) bladder,(33) prostate,(34) colon,(35) lung,(36) breast,(37) liver,(38) and renal cell cancer.(39) Mutations in TGFBR2, a putative tumor suppressor gene implicated in several malignancies, are also associated with inherited connective-tissue disorders.(40) The TGF-β/SMAD signaling pathway is constitutively activated in natural killer cells in acute lymphoblastic leukemia patients but not in healthy controls.(41) Functional variants of TGF-β1 genes may be significantly associated with the etiopathogenesis of acute myeloid leukemia.(42)

For peer review only - http://bmjopen.bmj.com/site/about/guidelines.xhtml

TGF-β1 also induces the PI3K/Akt/NF-κB signaling pathway during the recruitment of malignant cells in chronic myeloid leukemia.(43) A previous study performed in our laboratory has confirmed the association between malignancies and aortic aneurysms (unpublished data).

Limitations

The strength of our study is its population-based database design. We identified malignancies using the dual approaches of assessing ICD-9 code records and searching the registry of the Catastrophic Illness Patient Database to increase the accuracy of our data. We excluded confounding factors of malignancy, including comorbidities. Although we extensively adjusted our results by utilizing multivariate logistic regression models, our study nonetheless exhibited several limitations and did not address certain confounders. First, MFS might be underdiagnosed in Asian populations.(44) MFS is diagnosed using the Ghent criteria, which are primarily based on clinical features of Caucasian MFS populations; however, clinical features of the cardiovascular, ocular and skeletal systems significantly differ between Caucasian and Asian MFS populations.(44-46) Second, the NHIRD registry could not provide detailed information regarding laboratory results, family histories and health-related lifestyle factors, such as alcohol consumption or tobacco use, that can increase the risk

For peer review only - http://bmjopen.bmj.com/site/about/guidelines.xhtml

of malignancy and were potential confounding factors in this study. In our investigation, we also considered COPD incidence as a proxy variable for tobacco use to diminish the potential confounding effect of tobacco use on our results. (47) Third, this investigation was designed as a case-control study because this approach is efficient for relatively rare diseases or diseases with a long latency period, such as MFS, due to cost- and time-related considerations. Our study can identify associations between MFS and malignancies, but a case-control study cannot prove a cause-effect relationship. Ethnic differences in the association between MFS and malignancy should be further explored in larger, international, prospective follow-up studies enrolling multiple ethnic populations.

Conclusion

Patients with MFS are at increased risk of developing various malignancies. Health care professionals should be aware of this risk when treating patients with MFS. Relative to other patients, patients with MFS may require additional cancer surveillance.

Authors' contributors

C-WH and M-TL conceptualized and designed the study. J-CW, W-IL and S-HT

For peer review only - http://bmjopen.bmj.com/site/about/guidelines.xhtml

reviewed relevant literature and drafted the manuscript. W-CC provided the dataset and coordinated the study. C-HC, C-HT and Y-FW analyzed the data. All authors collected and interpreted the data and approved the manuscript.

Competing interests: None declared.

Data sharing statement: No additional data are available.

Acknowledgments

This study was supported by grants from Tri-Service General Hospital, National Defense Medical Center, Taipei, Taiwan (TSGH-C106-002; TSGH-C106-048); Taoyuan Armed Forces General Hospital, Taoyuan, Taiwan (10514); and the Ministry of Science and Technology (MOST 106-2314-B-016-031-).

For peer review only

Legends to tables and figures

Table 1 Baseline characteristics of the study population.

Table 2 Factors of Marfan syndrome determined using conditional logistic regression in a generalized estimating equation model.

Table 3 Analysis of the malignancy subgroup using conditional logistic regression in a generalized estimating equation model in patients with or without Marfan syndrome.

Table 4 Analysis of age and survival regarding malignancy in patients with or without Marfan syndrome.

Figure 1 Patient selection flowchart.

For peer review only - http://bmjopen.bmj.com/site/about/guidelines.xhtml

References

1. Halushka MK, Angelini A, Bartoloni G, Basso C, Batoroeva L, Bruneval P, et al. Consensus statement on surgical pathology of the aorta from the Society for Cardiovascular Pathology and the Association For European Cardiovascular Pathology: II. Noninflammatory degenerative diseases - nomenclature and diagnostic criteria. Cardiovascular pathology : the official journal of the Society for Cardiovascular Pathology. 2016;25(3):247-57.

2. Cook JR, Carta L, Galatioto J, Ramirez F. Cardiovascular manifestations in Marfan syndrome and related diseases; multiple genes causing similar phenotypes. Clinical genetics. 2015;87(1):11-20.

3. Kubiczkova L, Sedlarikova L, Hajek R, Sevcikova S. TGF-beta - an excellent servant but a bad master. Journal of translational medicine. 2012;10:183.

4. Katsuno Y, Lamouille S, Derynck R. TGF-beta signaling and epithelial-mesenchymal transition in cancer progression. Current opinion in oncology. 2013;25(1):76-84.

5. Corso A, Pagnucco G, Morra E, Bernasconi C. [The diagnosis of non-Hodgkin's lymphoma in a patient with Marfan's syndrome]. Minerva medica. 1993;84(7-8):417-9.

6. Sharief N, Kingston JE, Wright VM, Costeloe K. Acute leukemia in an infant with Marfan's syndrome: a case report. Pediatric hematology and oncology. 1991;8(4):323-7.

7. Miyajima Y, Kitase Y, Mizuno S, Sakai H, Matsumoto N, Ogawa A. [Acute lymphoblastic leukemia in a pediatric patient with Marfan's syndrome]. [Rinsho ketsueki] The Japanese journal of clinical hematology. 2011;52(1):28-31.

8. Winer DA, Winer S, Rotstein L, Asa SL, Mete O. Villous papillary thyroid carcinoma: a variant associated with marfan syndrome. Endocrine pathology. 2012;23(4):254-9.

9. Roopnariane A, Freed RJ, Price S, Fox EJ, Ritty TM. Osteosarcoma in a Marfan patient with a novel premature termination codon in the FBN1 gene. Connective tissue research. 2011;52(2):157-65.

10. Yoshitake K, Hagiwara Y, Tanae K, Takahashi N, Kohri M, Tamaru J, et al. [Marfan syndrome complicated with CD5+ CD10+ diffuse large B-cell lymphoma]. [Rinsho ketsueki] The Japanese journal of clinical hematology. 2010;51(3):196-200.

11. Lachanas E, Tomos P, Fotinou M, Miyakis S. Lymphoma presenting as chronic aortic dissection in Marfan syndrome. Annals of hematology. 2006;85(3):181-2.

12. Kraemer DM, Waschke J, Kunzmann V, Wilhelm M. Veno-occlusive disease in a male patient with Marfan syndrome and common acute lymphoblastic leukemia

For peer review only - http://bmjopen.bmj.com/site/about/guidelines.xhtml

during induction therapy. Annals of hematology. 2003;82(7):444-7.
13. Lee JJ, Kim HJ, Chung IJ, Jeong MH, Kook H, Seo JS, et al. A case of Marfan syndrome with acute monoblastic leukemia. The Korean journal of internal medicine. 1998;13(2):140-2.
14. Bisconti M, Bisetti A, Bidoli P. Malignant mesothelioma in subjects with Marfan's syndrome and Ehlers-Danlos syndrome: only an apparent association? Respiration; international review of thoracic diseases. 2000;67(2):223-8.
15. Togashi Y, Sakoda H, Sugahara H, Asagoe K, Matsuzawa Y. [Loeys-Dietz syndrome with acute myeloid leukemia]. [Rinsho ketsueki] The Japanese journal of clinical hematology. 2008;49(8):664-7.
16. Cheng CL, Kao YH, Lin SJ, Lee CH, Lai ML. Validation of the National Health Insurance Research Database with ischemic stroke cases in Taiwan. Pharmacoepidemiology and drug safety. 2011;20(3):236-42.
17. Mao CT, Tsai ML, Wang CY, Wen MS, Hsieh IC, Hung MJ, et al. Outcomes and characteristics of patients undergoing percutaneous angioplasty followed by below-knee or above-knee amputation for peripheral artery disease. PloS one. 2014;9(10):e111130.
18. Lee LJ-H, Chang Y-Y, Liou S-H, Wang J-D. Estimation of benefit of prevention of occupational cancer for comparative risk assessment: methods and examples. Occupational and Environmental Medicine. 2012;69(8):582-6.
19. Wu CY, Chan FK, Wu MS, Kuo KN, Wang CB, Tsao CR, et al. Histamine2-receptor antagonists are an alternative to proton pump inhibitor in patients receiving clopidogrel. Gastroenterology. 2010;139(4):1165-71.
20. Milleron O, Arnoult F, Ropers J, Aegerter P, Detaint D, Delorme G, et al. Marfan Sartan: a randomized, double-blind, placebo-controlled trial. European heart journal. 2015;36(32):2160-6.
21. Okada M, Takeuchi E, Mori Y, Ichihara S, Usui A, Ueda Y. An autopsy case of angiosarcoma arising around a woven Dacron prosthesis after a Cabrol operation. The Journal of thoracic and cardiovascular surgery. 2004;127(6):1843-5.
22. Dexeus FH, Logothetis CJ, Chong C, Sella A, Ogden S. Genetic abnormalities in men with germ cell tumors. The Journal of urology. 1988;140(1):80-4.
23. Newbold SG, Shafer AD, Goodwin CD, Nanagas VN, Dimlich SH. Stage III Wilms' tumor of a solitary kidney in a patient with Marfan's syndrome: a 5-yr survival. Journal of pediatric surgery. 1982;17(6):841-2.
24. Fassbender WJ, Krohn-Grimberghe B, Gortz B, Litzlbauer D, Stracke H, Raue F, et al. Multiple endocrine neoplasia (MEN)--an overview and case report--patient with sporadic bilateral pheochromocytoma, hyperparathyroidism and marfanoid habitus. Anticancer research. 2000;20(6c):4877-87.

For peer review only - http://bmjopen.bmj.com/site/about/guidelines.xhtml

25. Sotos JG. Abraham Lincoln's marfanoid mother: the earliest known case of multiple endocrine neoplasia type 2B? Clinical dysmorphology. 2012;21(3):131-6.
26. Papadopoulos A, Ntaios G, Kaiafa G, Girtovitis F, Saouli Z, Kontoninas Z, et al. Veno-occlusive disease of the liver during induction therapy for acute lymphoblastic leukemia. International journal of hematology. 2008;88(4):441-2.
27. Bell D, Marshman D. Diffuse Large B Cell Lymphoma in a Prosthetic Aortic Graft. Heart, lung & circulation. 2017;26(2):e4-e6.
28. Ghosh S, Ghosh S, Kumar Ghosh S. Aortic aneurysm and non-Hodgkin's lymphoma in Marfan syndrome. Turkish journal of haematology : official journal of Turkish Society of Haematology. 2009;26(1):31-3.
29. Davis MR, Andersson R, Severin J, de Hoon M, Bertin N, Baillie JK, et al. Transcriptional profiling of the human fibrillin/LTBP gene family, key regulators of mesenchymal cell functions. Molecular genetics and metabolism. 2014;112(1):73-83.
30. Franken R, den Hartog AW, de Waard V, Engele L, Radonic T, Lutter R, et al. Circulating transforming growth factor-beta as a prognostic biomarker in Marfan syndrome. International journal of cardiology. 2013;168(3):2441-6.
31. Morissette R, Schoenhoff F, Xu Z, Shilane DA, Griswold BF, Chen W, et al. Transforming growth factor-beta and inflammation in vascular (type IV) Ehlers-Danlos syndrome. Circulation Cardiovascular genetics. 2014;7(1):80-8.
32. Liu JF, Mao L, Bu LL, Ma SR, Huang CF, Zhang WF, et al. C4.4A as a biomarker of head and neck squamous cell carcinoma and correlated with epithelial mesenchymal transition. American journal of cancer research. 2015;5(12):3505-15.
33. Liang Y, Zhu F, Zhang H, Chen D, Zhang X, Gao Q, et al. Conditional ablation of TGF-beta signaling inhibits tumor progression and invasion in an induced mouse bladder cancer model. Scientific reports. 2016;6:29479.
34. Korrodi-Gregorio L, Silva JV, Santos-Sousa L, Freitas MJ, Felgueiras J, Fardilha M. TGF-beta cascade regulation by PPP1 and its interactors -impact on prostate cancer development and therapy. Journal of cellular and molecular medicine. 2014;18(4):555-67.
35. Ramamoorthi G, Sivalingam N. Molecular mechanism of TGF-beta signaling pathway in colon carcinogenesis and status of curcumin as chemopreventive strategy. Tumour biology : the journal of the International Society for Oncodevelopmental Biology and Medicine. 2014;35(8):7295-305.
36. Jeon HS, Jen J. TGF-beta signaling and the role of inhibitory Smads in non-small cell lung cancer. Journal of thoracic oncology : official publication of the International Association for the Study of Lung Cancer. 2010;5(4):417-9.
37. Zarzynska JM. Two faces of TGF-beta1 in breast cancer. Mediators of inflammation. 2014;2014:141747.

For peer review only - http://bmjopen.bmj.com/site/about/guidelines.xhtml

38. Yoshida K, Murata M, Yamaguchi T, Matsuzaki K. TGF-beta/Smad signaling during hepatic fibro-carcinogenesis (review). International journal of oncology. 2014;45(4):1363-71.
39. Bostrom AK, Lindgren D, Johansson ME, Axelson H. Effects of TGF-beta signaling in clear cell renal cell carcinoma cells. Biochem Biophys Res Commun. 2013;435(1):126-33.
40. Mizuguchi T, Collod-Beroud G, Akiyama T, Abifadel M, Harada N, Morisaki T, et al. Heterozygous TGFBR2 mutations in Marfan syndrome. Nature genetics. 2004;36(8):855-60.
41. Rouce RH, Shaim H, Sekine T, Weber G, Ballard B, Ku S, et al. The TGF-beta/SMAD pathway is an important mechanism for NK cell immune evasion in childhood B-acute lymphoblastic leukemia. Leukemia. 2016;30(4):800-11.
42. Nursal AF, Pehlivan M, Sahin HH, Pehlivan S. The Associations of IL-6, IFN-gamma, TNF-alpha, IL-10, and TGF-beta1 Functional Variants with Acute Myeloid Leukemia in Turkish Patients. Genetic testing and molecular biomarkers. 2016;20(9):544-51.
43. Zhu B, Zhang J, Chen J, Li C, Wang X. Molecular biological characteristics of the recruitment of hematopoietic stem cells from bone marrow niche in chronic myeloid leukemia. International journal of clinical and experimental pathology. 2015;8(10):12595-607.
44. Franken R, den Hartog AW, van de Riet L, Timmermans J, Scholte AJ, van den Berg MP, et al. Clinical features differ substantially between Caucasian and Asian populations of Marfan syndrome. Circulation journal : official journal of the Japanese Circulation Society. 2013;77(11):2793-8.
45. Yoo EH, Woo H, Ki CS, Lee HJ, Kim DK, Kang IS, et al. Clinical and genetic analysis of Korean patients with Marfan syndrome: possible ethnic differences in clinical manifestation. Clinical genetics. 2010;77(2):177-82.
46. Lim AY, Song JS, Kim EK, Jang SY, Chung TY, Choi SH, et al. Clinical Characteristics of Marfan Syndrome in Korea. Korean circulation journal. 2016;46(6):841-5.
47. Yu TM, Chuang YW, Yu MC, Chen CH, Yang CK, Huang ST, et al. Risk of cancer in patients with polycystic kidney disease: a propensity-score matched analysis of a nationwide, population-based cohort study. The Lancet Oncology. 2016.

For peer review only - http://bmjopen.bmj.com/site/about/guidelines.xhtml

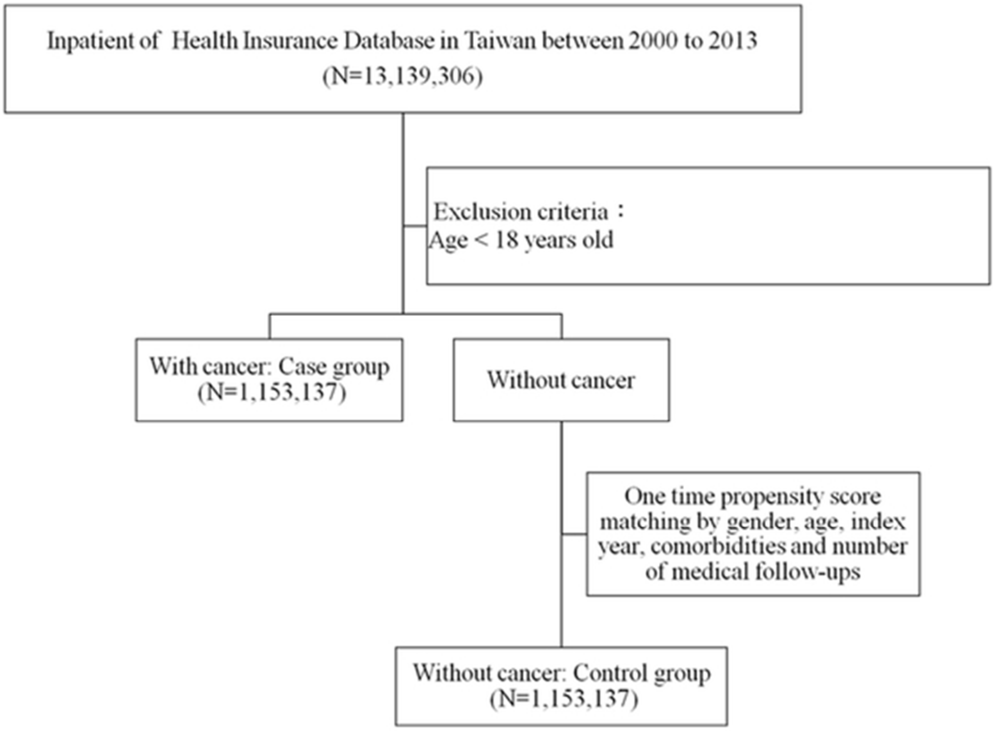

Figure 1

25x18mm (600 x 600 DPI)

STROBE 2007 (v4) Statement—Checklist of items that should be included in reports of *case-control studies*

Section/Topic	Item #	Recommendation	Reported on page #
**Title and abstract**	1	(*a*) Indicate the study's design with a commonly used term in the title or the abstract	1
		(*b*) Provide in the abstract an informative and balanced summary of what was done and what was found	2-3
**Introduction**			
Background/rationale	2	Explain the scientific background and rationale for the investigation being reported	4-5
Objectives	3	State specific objectives, including any prespecified hypotheses	6
**Methods**			
Study design	4	Present key elements of study design early in the paper	
Setting	5	Describe the setting, locations, and relevant dates, including periods of recruitment, exposure, follow-up, and data collection	6
Participants	6	(*a*) Give the eligibility criteria, and the sources and methods of case ascertainment and control selection. Give the rationale for the choice of cases and controls	7
		(*b*) For matched studies, give matching criteria and the number of controls per case	7-8
Variables	7	Clearly define all outcomes, exposures, predictors, potential confounders, and effect modifiers. Give diagnostic criteria, if applicable	8
Data sources/ measurement	8*	For each variable of interest, give sources of data and details of methods of assessment (measurement). Describe comparability of assessment methods if there is more than one group	
Bias	9	Describe any efforts to address potential sources of bias	
Study size	10	Explain how the study size was arrived at	10
Quantitative variables	11	Explain how quantitative variables were handled in the analyses. If applicable, describe which groupings were chosen and why	8-9
Statistical methods	12	(*a*) Describe all statistical methods, including those used to control for confounding	8-9
		(*b*) Describe any methods used to examine subgroups and interactions	n/a
		(*c*) Explain how missing data were addressed	n/a
		(*d*) If applicable, explain how matching of cases and controls was addressed	8
		(*e*) Describe any sensitivity analyses	n/a
**Results**			

For peer review only

Participants	13*	(a) Report numbers of individuals at each stage of study—eg numbers potentially eligible, examined for eligibility, confirmed eligible, included in the study, completing follow-up, and analysed	
		(b) Give reasons for non-participation at each stage	10
		(c) Consider use of a flow diagram	10
Descriptive data	14*	(a) Give characteristics of study participants (eg demographic, clinical, social) and information on exposures and potential confounders	10
		(b) Indicate number of participants with missing data for each variable of interest	n/a
Outcome data	15*	Report numbers in each exposure category, or summary measures of exposure	10
Main results	16	(*a*) Give unadjusted estimates and, if applicable, confounder-adjusted estimates and their precision (eg, 95% confidence interval). Make clear which confounders were adjusted for and why they were included	10
		(*b*) Report category boundaries when continuous variables were categorized	10
		(*c*) If relevant, consider translating estimates of relative risk into absolute risk for a meaningful time period	n/a
Other analyses	17	Report other analyses done—eg analyses of subgroups and interactions, and sensitivity analyses	n/a
**Discussion**			
Key results	18	Summarise key results with reference to study objectives	12
Limitations	19	Discuss limitations of the study, taking into account sources of potential bias or imprecision. Discuss both direction and magnitude of any potential bias	14
Interpretation	20	Give a cautious overall interpretation of results considering objectives, limitations, multiplicity of analyses, results from similar studies, and other relevant evidence	14
Generalisability	21	Discuss the generalisability (external validity) of the study results	15
**Other information**			
Funding	22	Give the source of funding and the role of the funders for the present study and, if applicable, for the original study on which the present article is based	15

*Give information separately for cases and controls in case-control studies and, if applicable, for exposed and unexposed groups in cohort and cross-sectional studies.

Note: An Explanation and Elaboration article discusses each checklist item and gives methodological background and published examples of transparent reporting. The STROBE checklist is best used in conjunction with this article (freely available on the Web sites of PLoS Medicine at http://www.plosmedicine.org/, Annals of Internal Medicine at http://www.annals.org/, and Epidemiology at http://www.epidem.com/). Information on the STROBE Initiative is available at www.strobe-statement.org.

For peer review only

BMJ Open

The association between malignancies and Marfan syndrome: a population-based, nested case-control study in Taiwan

Journal:	*BMJ Open*
Manuscript ID	bmjopen-2017-017243.R2
Article Type:	Research
Date Submitted by the Author:	09-Sep-2017
Complete List of Authors:	Hsu, Chin-Wang ; School of Medicine, College of Medicine, Taipei Medical University, Department of Emergency Medicine,<br>Wang, Jen-Chun; Tri-Service General Hospital, National Defensive Medical Center, department of Emergency Medicine<br>Liao, Wen-I; Emergency Medicine<br>Chien, Wu-Chien ; National Defense Medical Center<br>Chung, Chi-Hsiang; National Defense Medical Center<br>Tsao, Chang-Huei ; Tri-Service General Hospital, National Defensive Medical Center, department of Emergency Medicine, Department of Medical Research<br>Wu , Yung-Fu ; Tri-Service General Hospital, National Defense Medical Center<br>Liao, Min-Tser ; Taoyuan Armed Forces General Hospital,<br>Tsai, Shih-Hung; Tri-Service General Hospital, National Defense Medical Center, Department of Emeregncy Medicine
**Primary Subject Heading**:	Oncology
Secondary Subject Heading:	Genetics and genomics, Oncology
Keywords:	Marfan syndrome, Malignancy, fibrillin, transforming growth factor-β

SCHOLARONE™ Manuscripts

The association between malignancies and Marfan syndrome: a population-based, nested case-control study in Taiwan

Chin-Wang Hsu, MD [1,2], Jen-Chun Wang, MD [3,4], Wen-I Liao, MD [3], Wu-Chien Chien, PhD [5,6,*], Chi-Hsiang Chung, PhD [5,6,7], Chang-Huei Tsao, PhD [5], Yung-Fu Wu, PhD [5], Min-Tser Liao, MD[8], Shih-Hung Tsai, MD, PhD [3,*]

[1] Department of Emergency Medicine, School of Medicine, College of Medicine, Taipei Medical University, Taipei, Taiwan

[2] Department of Emergency and Critical Medicine, Wan Fang Hospital, Taipei Medical University, Taipei, Taiwan

[3] Department of Emergency Medicine, Tri-Service General Hospital, National Defense Medical Center, Taipei, Taiwan

[4] Institute of Clinical Medicine, National Yang-Ming University, Taipei, Taiwan

[5] Department of Medical Research, Tri-Service General Hospital, National Defense Medical Center, Taipei, Taiwan

[6] School of Public Health, National Defense Medical Center, Taipei, Taiwan

[7] Taiwanese Injury Prevention and Safety Promotion Association, Taipei, Taiwan

[8] Department of Pediatrics, Taoyuan Armed Forces General Hospital, Taoyuan, Taiwan

[*] W.-C. Chien and S.-H. Tsai contributed equally to this work and are co-corresponding authors.

*Corresponding authors:

Dr. Shih-Hung Tsai

Department of Emergency Medicine,

Tri-Service General Hospital, National Defense Medical Center, Taipei, Taiwan

No. 325, Sec. 2, Cheng-Kung Road, Neihu Dist., Taipei City 11490, Taiwan

Tel.: + 886-2-87923311-16877

Fax: + 886-2-87927034

E-mail: tsaishihung@yahoo.com.tw

Dr. Wu-Chien Chien

Department of Medical Research 7115R,

Tri-Service General Hospital, National, Defense Medical Center, No. 325, Section 2, Cheng-Kung Road, Neihu District, Taipei City, 11490, Taiwan

Tel.: + 886-2-87923100

E-mail: chienwu@mail.ndmctsgh.edu.tw

For peer review only - http://bmjopen.bmj.com/site/about/guidelines.xhtml

Abstract

Objective Marfan syndrome (MFS) involves a deficiency of the structural extracellular matrix component fibrillin-1 (FBN-1) and over-activation of the transforming growth factor-β (TGF-β) signaling pathway. The TGF-β signaling pathway also actively participates in malignant transformation. Although anecdotal case reports have suggested associations between MFS/MFS-like conditions and several hematological and solid malignancies, such associations have not been thoroughly evaluated in large-scale studies. We sought to use a nationwide health care insurance claim database to evaluate whether patients with MFS are at increased risk of malignancy.

Patients and Methods We conducted a nested case-control analysis using a database extracted from Taiwan's National Health Insurance Research Database (NHIRD). All medical conditions for each case and control were categorized using ICD-9 classifications. Odds ratios (ORs) and 95% confidence intervals (CIs) for associations between MFS and malignancies were estimated using conditional logistic regression and adjusted for comorbidities.

Results Our analyses included 1,153,137 cancer cases and 1,153,137 propensity score-matched controls. Relative to other subjects, patients with MFS had a significantly higher risk of having a malignancy (adjusted odds ratio (OR): 3.991) and

For peer review only - http://bmjopen.bmj.com/site/about/guidelines.xhtml
For peer review only

hypertension (adjusted OR: 1.964) and were significantly more likely to be male. Malignancies originating from the head and neck and the urinary tract were significantly more frequent among patients with MFS than among subjects without MFS.

Conclusion Patients with MFS are at increased risk of developing various malignancies. Health care professionals should be aware of this risk when treating such patients, and increased cancer surveillance may be necessary for these patients.

Key words: Marfan syndrome, malignancy, transforming growth factor-β, fibrillin

For peer review only

For peer review only - http://bmjopen.bmj.com/site/about/guidelines.xhtml

Strengths and limitations of this study

- The associations between MFS/MFS-like conditions and malignancies have not been thoroughly evaluated in large-scale studies. Thus, we used a nationwide healthcare insurance claim database to evaluate whether patients with MFS are at increased risk of malignancy. We found that patients with MFS are at increased risk of developing various malignancies.
- The NHIRD registry did not provide detailed information regarding laboratory results, family history and health-related lifestyle factors that could increase the risk of malignancy, and these factors represent potential confounding factors in this study.

For peer review only - http://bmjopen.bmj.com/site/about/guidelines.xhtml

Introduction

Marfan syndrome (MFS) is a pleiotropic connective tissue disease caused by a deficiency of the structural extracellular matrix component fibrillin-1 (FBN-1). The study of murine models of MFS has revealed the involvement of the transforming growth factor-β (TGF-β) signaling pathway in the pathogenesis of this disease. Overactivated TGF-β signaling is associated with MFS and several MFS-like conditions, including Loeys-Dietz syndrome, Shprintzen-Goldberg syndrome, aneurysm-osteoarthritis syndrome and syndromic thoracic aortic aneurysm. These conditions are also clearly associated with degenerative non-inflammatory structural cardiovascular diseases, including aortic root dilatation, thoracic aneurysm and aortic dissection.(1, 2) The TGF-β signaling pathway also actively participates in malignant transformation. In tumor cells, TGF-β loses its anti-proliferative response and becomes an oncogenic factor; as a result, TGF-β function is impaired in various solid and hematological malignancies.(3) TGF-β-induced epithelial-mesenchymal transition and reversion from mesenchymal to epithelial phenotypes contribute to the survival and dissemination of malignant cells.(4) Anecdotal case reports have suggested an association between MFS/MFS-like conditions and several hematological and solid malignancies.(5-15) However, associations between MFS/MFS-like conditions and malignancies have not been thoroughly evaluated in large-scale studies. In this respect,

For peer review only

we sought to use a nationwide health care insurance claim database to evaluate whether patients with MFS are at increased risk of malignancy.

For peer review only

For peer review only - http://bmjopen.bmj.com/site/about/guidelines.xhtml

Methods

Data source

Data for our nationwide, population-based, nested case-control study were obtained from inpatient care records and registration files from the Taiwan National Health Insurance Research Database (NHIRD). The National Health Insurance program was implemented in 1995 and provides health care coverage to 99% of the Taiwanese population (more than 23 million people). The accuracy of the NHIRD with respect to diagnoses for major diseases, such as stroke and acute coronary syndrome, has been validated.(16, 17) The confidentiality of individuals was protected by utilizing encrypted personal identification to avoid the possibility of ethical violations related to the study data. This investigation was conducted in accordance with the Declaration of Helsinki and other relevant guidelines. This study was approved by the Institutional Review Board of Tri-Service General Hospital, National Defense Medical Center, Taipei, Taiwan (TSGH IRB No. B-104-21).

Cancer cases and controls

This study involved a nested case-control design. Using the NHIRD, we selected adult patients >18 years of age who had been diagnosed with a malignancy based on ICD-9-DM codes (140-208) between 2000 and 2013 and confirmed these patients'

For peer review only

diagnoses by linking them to cases registered in the Catastrophic Illness Patient Database. The date of the first malignancy diagnosis was defined as the index date. We identified patients with MFS using the ICD-9-CM code 759.82. A sample of control candidates was selected for comparison from individuals in the NHIRD who were without malignancies. Patients in the study and control groups were selected via 1:1 matching by age, sex, number of medical follow-ups and comorbidities, including hypertension (ICD-9-CM 401-405), diabetes (ICD-9-CM 250), hyperlipidemia (ICD-9-CM 272.0-272.4), chronic obstructive pulmonary disease (COPD) (ICD-9-CM 490-496), alcoholism (ICD-9-CM 303), and obesity (ICD-9-CM 278). Data on the use of angiotensin-converting enzyme inhibitors and angiotensin receptor blockers were acquired from the Longitudinal Health Insurance Database 2005 (LHID 2005), a sub-database of the NHIRD. All insurance claims were scrutinized by medical reimbursement specialists, and peer review was undertaken according to standard and clinical diagnostic criteria, such as the pulmonary function test for COPD. Therefore, the diagnoses of COPD in this study should be highly reliable. Both the NHIRD and catastrophic illness certificate have been well-validated internally and externally in several studies.(16, 18, 19)

Outcome measurements

Only patients diagnosed with MFS prior to the index date were considered. MFS

For peer review only - http://bmjopen.bmj.com/site/about/guidelines.xhtml

was identified from the NHIRD by utilizing the corresponding ICD-9 code (ICD-9-CM code 759.82). The following covariates were included: age, gender, hypertension, diabetes mellitus (DM), hyperlipidemia, COPD, alcoholism and obesity.

Statistical analysis

Patients' clinical characteristics were expressed in numerical form. Categorical variables, which were presented as percentages, were compared using chi-square tests and Fisher's exact test. Continuous variables, which were presented as the mean and standard deviation, were compared using t-tests. The primary goal of this study was to determine whether a patient's clinical characteristics, such as MFS, were associated with malignancies. Associations between those outcomes (prognoses) and clinical characteristics were investigated using conditional logistic regression in a generalized estimating equation (GEE) model. The regression results are presented as adjusted odds ratios (ORs) with corresponding 95% confidence intervals (CIs). The threshold for statistical significance was $p<0.05$. All data analyses were conducted using SPSS version 18 (SPSS, Inc., Chicago, IL, USA).

For peer review only - http://bmjopen.bmj.com/site/about/guidelines.xhtml

Results

A flow diagram of the patient enrollment scheme is presented in **Figure 1**. During the study period, a total of 1,153,137 patients in follow-up after being diagnosed with a malignancy were identified in the NHIRD, which included a total of 13,139,306 inpatients. Other patients from the same database who did not have malignancies and were matched by age, gender, number of medical follow-ups and comorbidities were designated controls. As indicated in **Table 1**, as expected, there were no statistically significant differences between the case and control groups with respect to gender, age, number of medical follow-ups and comorbidities, including hypertension, DM, hyperlipidemia, COPD, alcoholism, and obesity, after matching.

Table 1 Baseline characteristics of the study population.

	Patients with malignancy N=1,153,137	Patients without malignancy N=1,153,137	*P* value
Marfan syndrome	93 (0.008%)	27 (0.002%)	<0.001
Gender			0.999
Male	657,188 (56.990%)	657,188 (56.990%)	
Female	495,969 (43.010%)	495,969 (43.010%)	
Age (years)	63.52±15.21	63.49±18.42	0.178
Comorbidities			
Hypertension	150,555 (13.056%)	150,512 (13.052%)	0.933
Diabetes	127,884 (11.090%)	127,894 (11.091%)	0.978
Hyperlipidemia	7,235 (0.627%)	7,233 (0.627%)	0.987
COPD	48,668 (4.220%)	48,656 (4.219%)	0.969
Alcoholism	1,052 (0.091%)	1,042 (0.090%)	0.827
Obesity	230 (0.020%)	247 (0.021%)	0.436

For peer review only

Medical follow-ups	4.84±10.70	4.82±10.87	0.159

COPD=chronic obstructive pulmonary disease.

At the end of the 13-year study period, the incidence of MFS was significantly higher among patients with malignancies compared with patients without malignancies over the corresponding observation period (0.008% vs. 0.003%, p<0.001). Among patients with MFS, there were no statistically significant differences regarding the use of angiotensin-converting enzyme inhibitors and angiotensin receptor blockers between patients with or without malignancies (20.93% vs 21.33%, p=0.95; 18.60% vs 19.11%, p=0.93, respectively). **Table 2** indicates that malignancy (adjusted OR=3.991, 95% CI: 2.555-6.235, p<0.001), maleness (adjusted OR=2.103, 95% CI: 1.427-3.098, p<0.001) and hypertension (adjusted OR=1.964, 95% CI: 1.258-3.064, p=0.013) were significantly associated with patients with MFS.

For peer review only

Table 2 Factors of Marfan syndrome determined using conditional logistic regression in a generalized estimating equation model.

	Crude OR*	95% CI	P value	Adjusted OR	95% CI	P value
Malignancy						
Without	Reference			Reference		
With	3.444	2.012-4.671	<0.001	3.999	2.578-6.301	<0.001
Gender						
Male	2.187	1.490-3.211	<0.001	2.110	1.429-3.101	<0.001
Female	Reference			Reference		
Hypertension						

Without	Reference			Reference		
With	1.503	1.013-2.324	0.001	1.962	1.224-3.000	0.010
Diabetes						
Without	Reference			Reference		
With	0.993	0.546-1.717	0.785	0.963	0.527-1.688	0.809
Hyperlipidemia						
Without	Reference			Reference		
With	0.748	0.197-2.862	0.704	0.711	0.214-3.201	0.764
COPD						
Without	Reference			Reference		
With	0.299	0.025-1.374	0.102	0.398	0.027-1.401	0.083
Alcoholism						
Without	Reference			Reference		
With	0.000	-	0.989	0.000	-	0.990
Obesity						
Without	Reference			Reference		
With	1.014	0.859-1.086	0.177	1.010	0.803-1.020	0.243

*OR=odds ratio; CI=confidence interval; Adjusted OR: adjusted by variables listed in the table.

As indicated in **Table 3**, malignancies originating from the head and neck (adjusted OR=8.714, 95% CI: 2.477-30.649, p<0.001), esophagus (adjusted OR=0.970, 95% CI: 3.943-30.522, p<0.001), stomach (adjusted OR=4.639, 95% CI: 1.743-12.345, p=0.002), colon and rectum (adjusted OR=3.134, 95% CI: 1.370-7.169, p=0.007), liver (adjusted OR=3.049, 95% CI: 1.349-6.980, p=0.007), female genital organs (adjusted OR=4.055, 95% CI: 1.417-11.606, p=0.009), prostate (adjusted OR=6.423, 95% CI: 2.355-17.517, p<0.001), urinary tract (adjusted OR=6.478, 95% CI: 2.974-14.112, p<0.001) and thyroid (adjusted OR=10.485, 95% CI: 4.251-25.861, p<0.001) and hematological malignancies (adjusted OR=6.981, 95% CI: 2.800-17.404,

For peer review only

p<0.001) were significantly more frequent among patients with MFS than among subjects without MFS.

Table 3 Analysis of malignancy subgroup using conditional logistic regression in a generalized estimating equation model in patients with or without Marfan syndrome.

Malignancy (with *vs.* without)	Adjusted OR	95% CI	P value
Total	3.999	2.578-6.301	<0.001
Head and neck	8.714	2.477-30.649	<0.001
Esophagus	10.970	3.943-30.522	<0.001
Stomach	4.639	1.743-12.345	0.002
Colon and rectum	3.134	1.370-7.169	0.007
Liver	3.049	1.349-6.980	0.007
Trachea, bronchus and lung	1.938	0.705-5.329	0.200
Female breast	0.477	0.060-3.782	0.483
Female genital organs	4.055	1.417-11.606	0.009
Prostate	6.423	2.355-17.517	<0.001
Bladder, ureter and kidney	6.478	2.974-14.112	<0.001
Thyroid	10.485	4.251-25.861	<0.001
Hematopoietic malignancy	6.981	2.800-17.404	<0.001
Others	2.900	1.434-5.863	0.003

Adjusted OR (odds ratio): adjusted for variables listed in Table 2; CI=confidence interval.

As indicated in **Table 4**, patients with MFS acquired malignancies at a younger age than those without MFS. During the study period, patients with MFS had a higher mortality rate and died younger than those without MFS..

Table 4 Analysis of age and survival regarding malignancy in patients with or without Marfan syndrome.

For peer review only

For peer review only - http://bmjopen.bmj.com/site/about/guidelines.xhtml

Marfan syndrome	With %	Without %	P value
Age at first inpatient visit due to malignancy	52.74±16.92	58.52±15.29	<0.001
Age at death during the follow-up period	67.35±1.63	70.84±11.85	<0.001
Patient mortality during the follow-up period	2.15%	0.197%	<0.001

For peer review only

Discussion

Using a nationwide population database, we have demonstrated for the first time that MFS is associated with the development of certain malignancies. No systemic approaches have previously been utilized to elucidate associations between MFS and malignancies.

Numerous case reports have addressed associations between MFS and various solid malignancies, including thyroid cancer,(8) osteosarcoma,(9) mesothelioma,(14) esophageal cancer,(20) angiosarcoma,(21) testicular cancer,(22) and Wilms' tumor.(23) Multiple endocrine neoplasia syndromes that are characterized by benign and malignant changes in multiple endocrine organs and incidental changes in nervous, muscular and connective tissues can also manifest marfanoid features.(24, 25) In our study, we observed that the frequencies of head and neck and urinary tract malignancies were significantly increased in patients with MFS. Accumulated case reports have also indicated that MFS and MFS-like conditions, such as Loeys-Dietz syndrome and Ehlers-Danlos syndrome (EDS), might be associated with various hematological malignancies and NHL.(5-7, 10-13, 15, 26)(27, 28) Larger, international studies should be conducted to study these potential associations.

For peer review only - http://bmjopen.bmj.com/site/about/guidelines.xhtml

As integral components of microfibrils, which provide strength and elasticity to the extracellular matrix, fibrillins, particularly FBN-1 and FBN-2, are thought to be involved in cancer pathogenesis and maintenance of the pluripotency of embryonic stem cells. Fibrillins are important for controlling the growth and differentiation of cells that they surround via interaction with integrins and growth factors as well as regulation of members of the TGF-β superfamily.(29) An FBN-1 deficiency impairs targeting of the large latent complex by the extracellular matrix, resulting in the unrestrained release of TGF-β ligands. An elevated TGF-β level in patients with MFS is correlated with larger aortic root diameters and faster aortic root growth.(30) Dysregulated TGF-β2 is also associated with vascular EDS.(31) TGF-β actively participates in malignant transformation and progression. Evidence has revealed associations between TGF-β and various solid malignancies, including head and neck,(32) bladder,(33) prostate,(34) colon,(35) lung,(36) breast,(37) liver,(38) and renal cell cancer.(39) Mutations in TGFBR2, a putative tumor suppressor gene implicated in several malignancies, are also associated with inherited connective-tissue disorders.(40) The TGF-β/SMAD signaling pathway is constitutively activated in natural killer cells in acute lymphoblastic leukemia patients but not in healthy controls.(41) Functional variants of TGF-β1 genes may be significantly associated with the etiopathogenesis of acute myeloid leukemia.(42)

For peer review only - http://bmjopen.bmj.com/site/about/guidelines.xhtml

TGF-β1 also induces the PI3K/Akt/NF-κB signaling pathway during the recruitment of malignant cells in chronic myeloid leukemia.(43) A previous study performed in our laboratory has confirmed the association between malignancies and aortic aneurysms (unpublished data).

Limitations

The strength of our study is its population-based database design. We identified malignancies using the dual approaches of assessing ICD-9 code records and searching the registry of the Catastrophic Illness Patient Database to increase the accuracy of our data. We excluded confounding factors of malignancy, including comorbidities. Although we extensively adjusted our results by utilizing multivariate logistic regression models, our study nonetheless exhibited several limitations and did not address certain confounders. First, MFS might be underdiagnosed in Asian populations.(44) MFS is diagnosed using the Ghent criteria, which are primarily based on clinical features of Caucasian MFS populations; however, clinical features of the cardiovascular, ocular and skeletal systems significantly differ between Caucasian and Asian MFS populations.(44-46) Second, the NHIRD registry could not provide detailed information regarding laboratory results, family histories and health-related lifestyle factors, such as alcohol consumption or tobacco use, that can increase the risk

For peer review only - http://bmjopen.bmj.com/site/about/guidelines.xhtml

of malignancy and were potential confounding factors in this study. In our investigation, we also considered COPD incidence as a proxy variable for tobacco use to diminish the potential confounding effect of tobacco use on our results. (47) Third, this investigation was designed as a case-control study because this approach is efficient for relatively rare diseases or diseases with a long latency period, such as MFS, due to cost- and time-related considerations. Our study can identify associations between MFS and malignancies, but a case-control study cannot prove a cause-effect relationship. Ethnic differences in the association between MFS and malignancy should be further explored in larger, international, prospective follow-up studies enrolling multiple ethnic populations.

Conclusion

Patients with MFS are at increased risk of developing various malignancies. Health care professionals should be aware of this risk when treating patients with MFS. Relative to other patients, patients with MFS may require additional cancer surveillance.

Authors' contributors

C-WH and M-TL conceptualized and designed the study. J-CW, W-IL and S-HT

For peer review only - http://bmjopen.bmj.com/site/about/guidelines.xhtml

reviewed relevant literature and drafted the manuscript. W-CC provided the dataset and coordinated the study. C-HC, C-HT and Y-FW analyzed the data. All authors collected and interpreted the data and approved the manuscript.

Competing interests: None declared.

Data sharing statement: No additional data are available.

Acknowledgments

This study was supported by grants from Tri-Service General Hospital, National Defense Medical Center, Taipei, Taiwan (TSGH-C106-002; TSGH-C106-048); Taoyuan Armed Forces General Hospital, Taoyuan, Taiwan (10514); and the Ministry of Science and Technology (MOST 106-2314-B-016-031-).

For peer review only - http://bmjopen.bmj.com/site/about/guidelines.xhtml

Legends to tables and figures

Table 1 Baseline characteristics of the study population.

Table 2 Factors of Marfan syndrome determined using conditional logistic regression in a generalized estimating equation model.

Table 3 Analysis of the malignancy subgroup using conditional logistic regression in a generalized estimating equation model in patients with or without Marfan syndrome.

Table 4 Analysis of age and survival regarding malignancy in patients with or without Marfan syndrome.

Figure 1 Patient selection flowchart.

For peer review only

References

1. Halushka MK, Angelini A, Bartoloni G, Basso C, Batoroeva L, Bruneval P, et al. Consensus statement on surgical pathology of the aorta from the Society for Cardiovascular Pathology and the Association For European Cardiovascular Pathology: II. Noninflammatory degenerative diseases - nomenclature and diagnostic criteria. Cardiovascular pathology : the official journal of the Society for Cardiovascular Pathology. 2016;25(3):247-57.

2. Cook JR, Carta L, Galatioto J, Ramirez F. Cardiovascular manifestations in Marfan syndrome and related diseases; multiple genes causing similar phenotypes. Clinical genetics. 2015;87(1):11-20.

3. Kubiczkova L, Sedlarikova L, Hajek R, Sevcikova S. TGF-beta - an excellent servant but a bad master. Journal of translational medicine. 2012;10:183.

4. Katsuno Y, Lamouille S, Derynck R. TGF-beta signaling and epithelial-mesenchymal transition in cancer progression. Current opinion in oncology. 2013;25(1):76-84.

5. Corso A, Pagnucco G, Morra E, Bernasconi C. [The diagnosis of non-Hodgkin's lymphoma in a patient with Marfan's syndrome]. Minerva medica. 1993;84(7-8):417-9.

6. Sharief N, Kingston JE, Wright VM, Costeloe K. Acute leukemia in an infant with Marfan's syndrome: a case report. Pediatric hematology and oncology. 1991;8(4):323-7.

7. Miyajima Y, Kitase Y, Mizuno S, Sakai H, Matsumoto N, Ogawa A. [Acute lymphoblastic leukemia in a pediatric patient with Marfan's syndrome]. [Rinsho ketsueki] The Japanese journal of clinical hematology. 2011;52(1):28-31.

8. Winer DA, Winer S, Rotstein L, Asa SL, Mete O. Villous papillary thyroid carcinoma: a variant associated with marfan syndrome. Endocrine pathology. 2012;23(4):254-9.

9. Roopnariane A, Freed RJ, Price S, Fox EJ, Ritty TM. Osteosarcoma in a Marfan patient with a novel premature termination codon in the FBN1 gene. Connective tissue research. 2011;52(2):157-65.

10. Yoshitake K, Hagiwara Y, Tanae K, Takahashi N, Kohri M, Tamaru J, et al. [Marfan syndrome complicated with CD5+ CD10+ diffuse large B-cell lymphoma]. [Rinsho ketsueki] The Japanese journal of clinical hematology. 2010;51(3):196-200.

11. Lachanas E, Tomos P, Fotinou M, Miyakis S. Lymphoma presenting as chronic aortic dissection in Marfan syndrome. Annals of hematology. 2006;85(3):181-2.

12. Kraemer DM, Waschke J, Kunzmann V, Wilhelm M. Veno-occlusive disease in a male patient with Marfan syndrome and common acute lymphoblastic leukemia

For peer review only - http://bmjopen.bmj.com/site/about/guidelines.xhtml

during induction therapy. Annals of hematology. 2003;82(7):444-7.

13. Lee JJ, Kim HJ, Chung IJ, Jeong MH, Kook H, Seo JS, et al. A case of Marfan syndrome with acute monoblastic leukemia. The Korean journal of internal medicine. 1998;13(2):140-2.

14. Bisconti M, Bisetti A, Bidoli P. Malignant mesothelioma in subjects with Marfan's syndrome and Ehlers-Danlos syndrome: only an apparent association? Respiration; international review of thoracic diseases. 2000;67(2):223-8.

15. Togashi Y, Sakoda H, Sugahara H, Asagoe K, Matsuzawa Y. [Loeys-Dietz syndrome with acute myeloid leukemia]. [Rinsho ketsueki] The Japanese journal of clinical hematology. 2008;49(8):664-7.

16. Cheng CL, Kao YH, Lin SJ, Lee CH, Lai ML. Validation of the National Health Insurance Research Database with ischemic stroke cases in Taiwan. Pharmacoepidemiology and drug safety. 2011;20(3):236-42.

17. Mao CT, Tsai ML, Wang CY, Wen MS, Hsieh IC, Hung MJ, et al. Outcomes and characteristics of patients undergoing percutaneous angioplasty followed by below-knee or above-knee amputation for peripheral artery disease. PloS one. 2014;9(10):e111130.

18. Lee LJ-H, Chang Y-Y, Liou S-H, Wang J-D. Estimation of benefit of prevention of occupational cancer for comparative risk assessment: methods and examples. Occupational and Environmental Medicine. 2012;69(8):582-6.

19. Wu CY, Chan FK, Wu MS, Kuo KN, Wang CB, Tsao CR, et al. Histamine2-receptor antagonists are an alternative to proton pump inhibitor in patients receiving clopidogrel. Gastroenterology. 2010;139(4):1165-71.

20. Milleron O, Arnoult F, Ropers J, Aegerter P, Detaint D, Delorme G, et al. Marfan Sartan: a randomized, double-blind, placebo-controlled trial. European heart journal. 2015;36(32):2160-6.

21. Okada M, Takeuchi E, Mori Y, Ichihara S, Usui A, Ueda Y. An autopsy case of angiosarcoma arising around a woven Dacron prosthesis after a Cabrol operation. The Journal of thoracic and cardiovascular surgery. 2004;127(6):1843-5.

22. Dexeus FH, Logothetis CJ, Chong C, Sella A, Ogden S. Genetic abnormalities in men with germ cell tumors. The Journal of urology. 1988;140(1):80-4.

23. Newbold SG, Shafer AD, Goodwin CD, Nanagas VN, Dimlich SH. Stage III Wilms' tumor of a solitary kidney in a patient with Marfan's syndrome: a 5-yr survival. Journal of pediatric surgery. 1982;17(6):841-2.

24. Fassbender WJ, Krohn-Grimberghe B, Gortz B, Litzlbauer D, Stracke H, Raue F, et al. Multiple endocrine neoplasia (MEN)--an overview and case report--patient with sporadic bilateral pheochromocytoma, hyperparathyroidism and marfanoid habitus. Anticancer research. 2000;20(6c):4877-87.

For peer review only - http://bmjopen.bmj.com/site/about/guidelines.xhtml

25. Sotos JG. Abraham Lincoln's marfanoid mother: the earliest known case of multiple endocrine neoplasia type 2B? Clinical dysmorphology. 2012;21(3):131-6.
26. Papadopoulos A, Ntaios G, Kaiafa G, Girtovitis F, Saouli Z, Kontoninas Z, et al. Veno-occlusive disease of the liver during induction therapy for acute lymphoblastic leukemia. International journal of hematology. 2008;88(4):441-2.
27. Bell D, Marshman D. Diffuse Large B Cell Lymphoma in a Prosthetic Aortic Graft. Heart, lung & circulation. 2017;26(2):e4-e6.
28. Ghosh S, Ghosh S, Kumar Ghosh S. Aortic aneurysm and non-Hodgkin's lymphoma in Marfan syndrome. Turkish journal of haematology : official journal of Turkish Society of Haematology. 2009;26(1):31-3.
29. Davis MR, Andersson R, Severin J, de Hoon M, Bertin N, Baillie JK, et al. Transcriptional profiling of the human fibrillin/LTBP gene family, key regulators of mesenchymal cell functions. Molecular genetics and metabolism. 2014;112(1):73-83.
30. Franken R, den Hartog AW, de Waard V, Engele L, Radonic T, Lutter R, et al. Circulating transforming growth factor-beta as a prognostic biomarker in Marfan syndrome. International journal of cardiology. 2013;168(3):2441-6.
31. Morissette R, Schoenhoff F, Xu Z, Shilane DA, Griswold BF, Chen W, et al. Transforming growth factor-beta and inflammation in vascular (type IV) Ehlers-Danlos syndrome. Circulation Cardiovascular genetics. 2014;7(1):80-8.
32. Liu JF, Mao L, Bu LL, Ma SR, Huang CF, Zhang WF, et al. C4.4A as a biomarker of head and neck squamous cell carcinoma and correlated with epithelial mesenchymal transition. American journal of cancer research. 2015;5(12):3505-15.
33. Liang Y, Zhu F, Zhang H, Chen D, Zhang X, Gao Q, et al. Conditional ablation of TGF-beta signaling inhibits tumor progression and invasion in an induced mouse bladder cancer model. Scientific reports. 2016;6:29479.
34. Korrodi-Gregorio L, Silva JV, Santos-Sousa L, Freitas MJ, Felgueiras J, Fardilha M. TGF-beta cascade regulation by PPP1 and its interactors -impact on prostate cancer development and therapy. Journal of cellular and molecular medicine. 2014;18(4):555-67.
35. Ramamoorthi G, Sivalingam N. Molecular mechanism of TGF-beta signaling pathway in colon carcinogenesis and status of curcumin as chemopreventive strategy. Tumour biology : the journal of the International Society for Oncodevelopmental Biology and Medicine. 2014;35(8):7295-305.
36. Jeon HS, Jen J. TGF-beta signaling and the role of inhibitory Smads in non-small cell lung cancer. Journal of thoracic oncology : official publication of the International Association for the Study of Lung Cancer. 2010;5(4):417-9.
37. Zarzynska JM. Two faces of TGF-beta1 in breast cancer. Mediators of inflammation. 2014;2014:141747.

For peer review only

38. Yoshida K, Murata M, Yamaguchi T, Matsuzaki K. TGF-beta/Smad signaling during hepatic fibro-carcinogenesis (review). International journal of oncology. 2014;45(4):1363-71.

39. Bostrom AK, Lindgren D, Johansson ME, Axelson H. Effects of TGF-beta signaling in clear cell renal cell carcinoma cells. Biochem Biophys Res Commun. 2013;435(1):126-33.

40. Mizuguchi T, Collod-Beroud G, Akiyama T, Abifadel M, Harada N, Morisaki T, et al. Heterozygous TGFBR2 mutations in Marfan syndrome. Nature genetics. 2004;36(8):855-60.

41. Rouce RH, Shaim H, Sekine T, Weber G, Ballard B, Ku S, et al. The TGF-beta/SMAD pathway is an important mechanism for NK cell immune evasion in childhood B-acute lymphoblastic leukemia. Leukemia. 2016;30(4):800-11.

42. Nursal AF, Pehlivan M, Sahin HH, Pehlivan S. The Associations of IL-6, IFN-gamma, TNF-alpha, IL-10, and TGF-beta1 Functional Variants with Acute Myeloid Leukemia in Turkish Patients. Genetic testing and molecular biomarkers. 2016;20(9):544-51.

43. Zhu B, Zhang J, Chen J, Li C, Wang X. Molecular biological characteristics of the recruitment of hematopoietic stem cells from bone marrow niche in chronic myeloid leukemia. International journal of clinical and experimental pathology. 2015;8(10):12595-607.

44. Franken R, den Hartog AW, van de Riet L, Timmermans J, Scholte AJ, van den Berg MP, et al. Clinical features differ substantially between Caucasian and Asian populations of Marfan syndrome. Circulation journal : official journal of the Japanese Circulation Society. 2013;77(11):2793-8.

45. Yoo EH, Woo H, Ki CS, Lee HJ, Kim DK, Kang IS, et al. Clinical and genetic analysis of Korean patients with Marfan syndrome: possible ethnic differences in clinical manifestation. Clinical genetics. 2010;77(2):177-82.

46. Lim AY, Song JS, Kim EK, Jang SY, Chung TY, Choi SH, et al. Clinical Characteristics of Marfan Syndrome in Korea. Korean circulation journal. 2016;46(6):841-5.

47. Yu TM, Chuang YW, Yu MC, Chen CH, Yang CK, Huang ST, et al. Risk of cancer in patients with polycystic kidney disease: a propensity-score matched analysis of a nationwide, population-based cohort study. The Lancet Oncology. 2016.

For peer review only - http://bmjopen.bmj.com/site/about/guidelines.xhtml

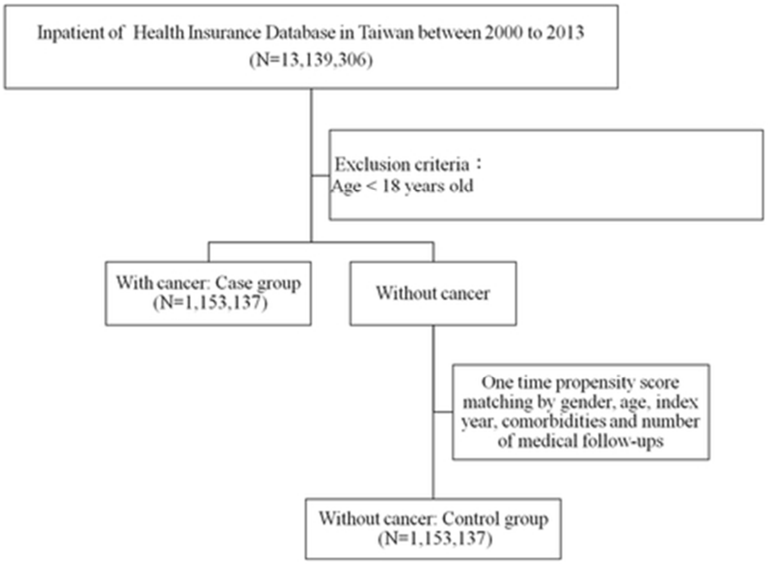

25x14mm (600 x 600 DPI)

For peer review only

STROBE 2007 (v4) Statement—Checklist of items that should be included in reports of *case-control studies*

Section/Topic	Item #	Recommendation	Reported on page #
**Title and abstract**	1	(*a*) Indicate the study's design with a commonly used term in the title or the abstract	1
		(*b*) Provide in the abstract an informative and balanced summary of what was done and what was found	2-3
**Introduction**			
Background/rationale	2	Explain the scientific background and rationale for the investigation being reported	4-5
Objectives	3	State specific objectives, including any prespecified hypotheses	6
**Methods**			
Study design	4	Present key elements of study design early in the paper	
Setting	5	Describe the setting, locations, and relevant dates, including periods of recruitment, exposure, follow-up, and data collection	6
Participants	6	(*a*) Give the eligibility criteria, and the sources and methods of case ascertainment and control selection. Give the rationale for the choice of cases and controls	7
		(*b*) For matched studies, give matching criteria and the number of controls per case	7-8
Variables	7	Clearly define all outcomes, exposures, predictors, potential confounders, and effect modifiers. Give diagnostic criteria, if applicable	8
Data sources/ measurement	8*	For each variable of interest, give sources of data and details of methods of assessment (measurement). Describe comparability of assessment methods if there is more than one group	
Bias	9	Describe any efforts to address potential sources of bias	
Study size	10	Explain how the study size was arrived at	10
Quantitative variables	11	Explain how quantitative variables were handled in the analyses. If applicable, describe which groupings were chosen and why	8-9
Statistical methods	12	(*a*) Describe all statistical methods, including those used to control for confounding	8-9
		(*b*) Describe any methods used to examine subgroups and interactions	n/a
		(*c*) Explain how missing data were addressed	n/a
		(*d*) If applicable, explain how matching of cases and controls was addressed	8
		(*e*) Describe any sensitivity analyses	n/a
**Results**			

For peer review only

Participants	13*	(a) Report numbers of individuals at each stage of study—eg numbers potentially eligible, examined for eligibility, confirmed eligible, included in the study, completing follow-up, and analysed	
		(b) Give reasons for non-participation at each stage	10
		(c) Consider use of a flow diagram	10
Descriptive data	14*	(a) Give characteristics of study participants (eg demographic, clinical, social) and information on exposures and potential confounders	10
		(b) Indicate number of participants with missing data for each variable of interest	n/a
Outcome data	15*	Report numbers in each exposure category, or summary measures of exposure	10
Main results	16	(*a*) Give unadjusted estimates and, if applicable, confounder-adjusted estimates and their precision (eg, 95% confidence interval). Make clear which confounders were adjusted for and why they were included	10
		(*b*) Report category boundaries when continuous variables were categorized	10
		(*c*) If relevant, consider translating estimates of relative risk into absolute risk for a meaningful time period	n/a
Other analyses	17	Report other analyses done—eg analyses of subgroups and interactions, and sensitivity analyses	n/a
**Discussion**			
Key results	18	Summarise key results with reference to study objectives	12
Limitations	19	Discuss limitations of the study, taking into account sources of potential bias or imprecision. Discuss both direction and magnitude of any potential bias	14
Interpretation	20	Give a cautious overall interpretation of results considering objectives, limitations, multiplicity of analyses, results from similar studies, and other relevant evidence	14
Generalisability	21	Discuss the generalisability (external validity) of the study results	15
**Other information**			
Funding	22	Give the source of funding and the role of the funders for the present study and, if applicable, for the original study on which the present article is based	15

*Give information separately for cases and controls in case-control studies and, if applicable, for exposed and unexposed groups in cohort and cross-sectional studies.

Note: An Explanation and Elaboration article discusses each checklist item and gives methodological background and published examples of transparent reporting. The STROBE checklist is best used in conjunction with this article (freely available on the Web sites of PLoS Medicine at http://www.plosmedicine.org/, Annals of Internal Medicine at http://www.annals.org/, and Epidemiology at http://www.epidem.com/). Information on the STROBE Initiative is available at www.strobe-statement.org.

For peer review only
